# Supplementary material for: A slow transcription rate causes embryonic lethality and perturbs kinetic coupling of neuronal genes
Source: EMBO J. 2019 Apr 15;38(9):e101244. doi: 10.15252/embj.2018101244 (PMC6484407; doi:10.15252/embj.2018101244)
Supplement: Supplementary file 1 — Appendix [file EMBJ-38-e101244-s001.docx]

**Appendix for**

**A slow transcription rate causes embryonic lethality and perturbs kinetic coupling of neuronal genes**

**Magdalena M. Maslon^1^, Ulrich Braunschweig^2^, Stuart Aitken^1^,**

**Abigail R. Mann^1^, Fiona Kilanowski^1^, Chris J. Hunter^1^,**

**Benjamin J. Blencowe^2^, Alberto R. Kornblihtt^3^, Ian R. Adams^1*^&**

**Javier F. Cáceres^1*^**

^1^MRC Human Genetics Unit, Institute of Genetics and Molecular Medicine, University of Edinburgh, UK; ^2^Donnelly Centre, University of Toronto, Ontario M5S 3E1, Canada; ^3^Instituto de Fisiología, Biología Molecular y Neurociencias (IFIBYNE-UBA-CONICET) and Departamento de Fisiología, Biología Molecular y Celular, Facultad de Ciencias Exactas y Naturales, Universidad de Buenos Aires, Ciudad Universitaria, C1428EHA Buenos Aires, Argentina.

**Table of contents for Appendix Files**

**Appendix Figure S1.**

**Appendix Figure S2.**

**Appendix Figure S3.**

**Appendix Figure S4.**

**Appendix Figure S5.**

**Appendix Figure S6.**

**Appendix References**

**Appendix Table S1**

**Appendix Table S2**

**Appendix Figures**

**Appendix Figure S1 (related to Fig. 4)**

A Gene ontology analysis of genes upregulated in slow/slow NPCs.

B RT-qPCR showing differential expression of selected genes in ESCs (d0), at day 3 of monolayer differentiation, in NPCs (d7) and AGGs. Data are represented as a fold change relative to ESC expression ± standard error, n=3.

**Appendix Figure S2 (related to Fig. 5)**

A RNA-binding proteins (RBPs), which display a change in expression throughtout different stages of differentiation. Upregulation and downregulation of RBPs is highlighted by red and blue color, respectively, whilst numbers represent Log2FC.

B       The number of differentially spliced exons in slow/slow cells that overlap with the targets of the indicated RBPs is indicated. The corresponding total number of targets identified in these studies is indicated in brackets . Available RNA-seq data from splicing factor knockdowns or knockouts and their associated controls that was used include: MBNL2 in mouse hippocampi (1,2), Ptbp2 in mouse brain (3), HNRNPA2B1 in mouse spinal cord (4), KHDRSB3 in mouse brain (5), MBNL3 in C2C12 myoblasts (6), Nova1   in mouse brain   (7,8). The corresponding total number of targets identified in respective studies is indicated in brackets. Numbers in superscript represent the references, as listed in the Appendix references.

**Appendix References**

1. Goodwin M, Mohan A, Batra R, Lee K-Y, Charizanis K, Gómez FJF, Eddarkaoui S, Sergeant N, Buée L, Kimura T, et al. 2015. MBNL Sequestration by Toxic RNAs and RNA Misprocessing in the Myotonic Dystrophy Brain. *Cell Rep* **12**: 1159–1168.

2. Charizanis K, Lee K-Y, Batra R, Goodwin M, Zhang C, Yuan Y, Shiue L, Cline M, Scotti MM, Xia G, et al. 2012. Muscleblind-like 2-mediated alternative splicing in the developing brain and dysregulation in myotonic dystrophy. *Neuron* **75**: 437–50.

3. Li Q, Zheng S, Han A, Lin C-H, Stoilov P, Fu X-D, Black DL. 2014. The splicing regulator PTBP2 controls a program of embryonic splicing required for neuronal maturation. *Elife* **3**: e01201.

4. Martinez FJ, Pratt GA, Van Nostrand EL, Batra R, Huelga SC, Kapeli K, Freese P, Chun SJ, Ling K, Gelboin-Burkhart C, et al. 2016. Protein-RNA Networks Regulated by Normal and ALS-Associated Mutant HNRNPA2B1 in the Nervous System. *Neuron* **92**: 780–795.

5. Ehrmann I, Dalgliesh C, Liu Y, Danilenko M, Crosier M, Overman L, Arthur HM, Lindsay S, Clowry GJ, Venables JP, et al. 2013. The tissue-specific RNA binding protein T-STAR controls regional splicing patterns of neurexin pre-mRNAs in the brain. *PLoS Genet* **9**: e1003474.

6. Poulos MG, Batra R, Li M, Yuan Y, Zhang C, Darnell RB, Swanson MS. 2013. Progressive impairment of muscle regeneration in muscleblind-like 3 isoform knockout mice. *Hum Mol Genet* **22**: 3547–58.

7. Ule J, Stefani G, Mele A, Ruggiu M, Wang X, Taneri B, Gaasterland T, Blencowe BJ, Darnell RB. 2006. An RNA map predicting Nova-dependent splicing regulation. *Nature* **444**: 580–586.

8. Zhang C, Frias MA, Mele A, Ruggiu M, Eom T, Marney CB, Wang H, Licatalosi DD, Fak JJ, Darnell RB. 2010. Integrative modeling defines the Nova splicing-regulatory network and its combinatorial controls. *Science* **329**: 439–43.

**Appendix Figure S3 (related to Fig. 5).**

RT-PCR analysis validation of selected alternatively spliced exons. RT-PCR was performed on total RNA from WT or slow/slow ESCs, NPCs or neurons. PCR products were visualized and quantified by Bioanalyzer (Agilent). Images are representative of experiments performed in triplicate.

**Appendix Figure S4 (related to Fig. 5). A** **reduced transcriptional elongation results in gross changes in gene expression during neural differentiation.**

A MA plots for pluripotent ESCs and all stages of neural differentiation. The y- axis depicts the log_2_ fold change in gene expression in slow/slow cells (M), as compared to WT, whereas the x-axis is the average of log_2_ mean read count for each gene between all samples. Each dot represents one gene. Red dots depict genes with significantly altered expression values (Benjamini and Hochberg (B + H) adjusted *p* value <0.01), with mean expression of at least RPKM=5. Grey line depicts fold change (FC) of either two-fold upregulation or downregulation.

B Number of upregulated and downregulated genes in ESCs and at different stages of neural differentiation.

**Appendix Figure S5 (related to Fig. 6).**

Cytoscape-generated enrichment map, visualizing the results of gene-sets enrichment for genes downregulated and differentially spliced in slow/slow neurons as a network, where nodes represent the gene-sets, and edges their similarity, size of nodes represents number of genes.

**Appendix Figure S6 (related to Figs. 5 and Fig. 6).**

Heatmaps displaying the correlation of gene expression (A) and dPSI (B) for all pairwise combinations of samples in the dataset.

**Appendix Tables**

| **Co-expressed in ESCs and NPCs** | | |
| --- | --- | --- |
| AS/ESCs only | AS/NPCs only |  |
| 104 | 946 |  |
| **Co-expressed in ESCs and neurons** | | |
| AS/ESCs only | AS/neurons only |  |
| 150 | 730 |  |
| **Co-expressed in ALL** | | |
| AS/ESCs only | AS/NPCs only | AS/neurons only |
| 66 | 751 | 610 |

**Appendix Table S1.** Number of Slow RNAPII regulated alternative splicing events for genes that are expressed in both or all three stages of differentiation, but differentially spliced only in one of them.

|  | **Matched (Total)** | **p-value** |
| --- | --- | --- |
| ASD (SFARI) | 77 (1008) | 2.68E-05 |
| Schizophrenia | 25 (240) | 0.000152 |
| Neuroblastoma | 67 (1003) | 0.00237 |

**Appendix Table S2.** Overlap of slow RNAPII regulated genes in neurons with genes ascribed to ASD, schizophrenia, neuroblastoma, including results of Fishers test.
