# Supplementary material for: A slow transcription rate causes embryonic lethality and perturbs kinetic coupling of neuronal genes
Source: EMBO J. 2019 Apr 15;38(9):e101244. doi: 10.15252/embj.2018101244 (PMC6484407; doi:10.15252/embj.2018101244)
Supplement: Supplementary file 2 — Expanded View Figures PDF [file EMBJ-38-e101244-s002.pdf]

Expanded View Figures

Figure EV1. Validation of the transcriptional elongation rate in ES mutant cell lines (related to Fig 3).

A Cartoon depicting the structure of Itpr1 and utrophin pre-mRNAs, with primer pairs selected to monitor the appearance of selected intron–exon junctions indicated by arrows. Quantification of pre-mRNA at different time points at the beginning, in the middle, and at the end of the gene displayed relative to cells not treated with DRB in WT/WT, WT/slow, and slow/slow cells (black, gray, and purple bars, respectively). The mean ± SEM is shown, $n = 3$.

B Time course of incorporation of ^{3}H-uridine in WT/WT or slow/slow cells. Time 0' corresponds to time of DRB wash-off. Mean ± SEM is plotted with $*P < 0.05$ as determined by t-test, $n = 3$.

C *In vitro* transcription of a linearized plasmid using nuclear extracts from either WT/WT or slow/slow ES cells. The image shown is representative of 1 of 2 reproducible experiments.

© 2019 The Authors

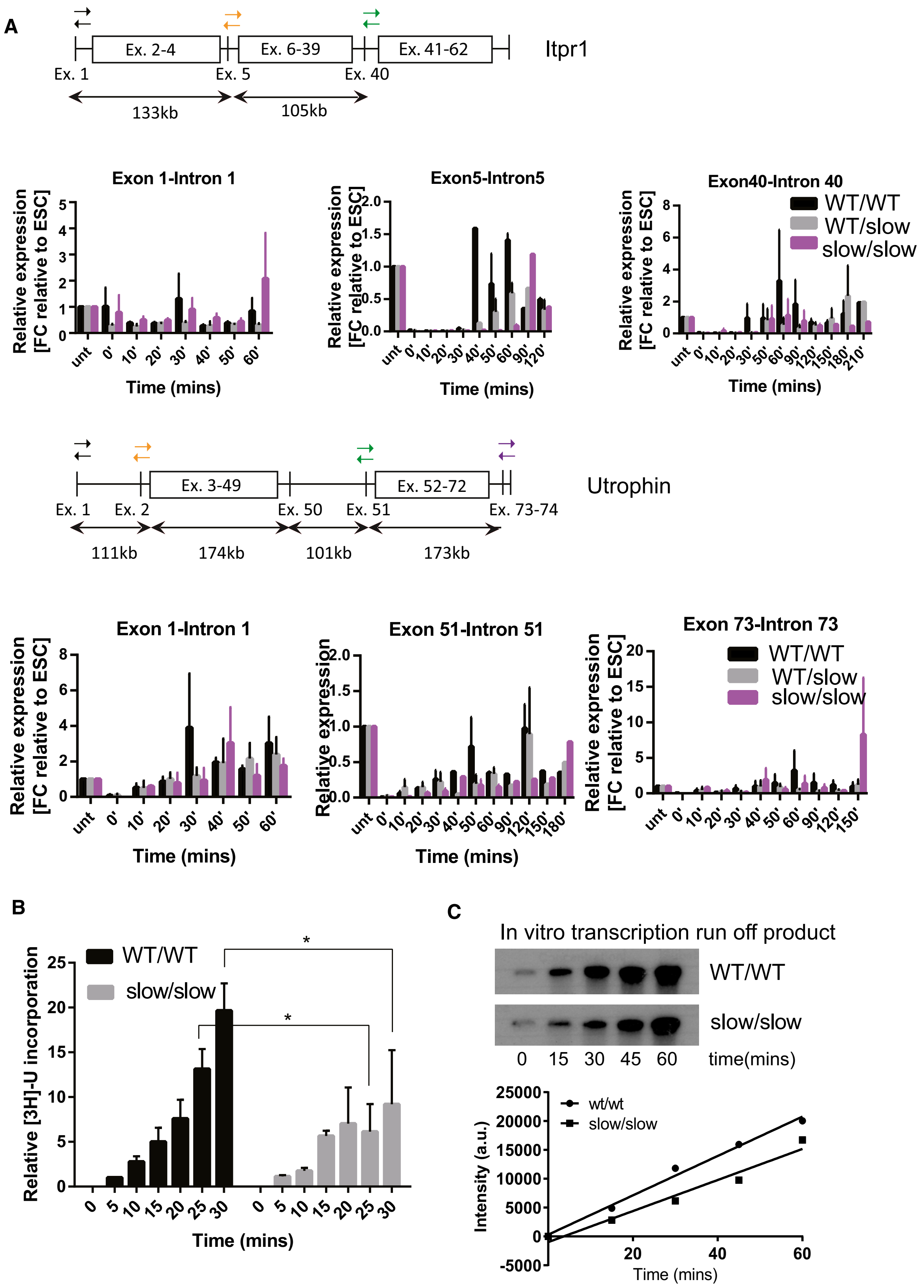

Figure EV1.

© 2019 The Authors

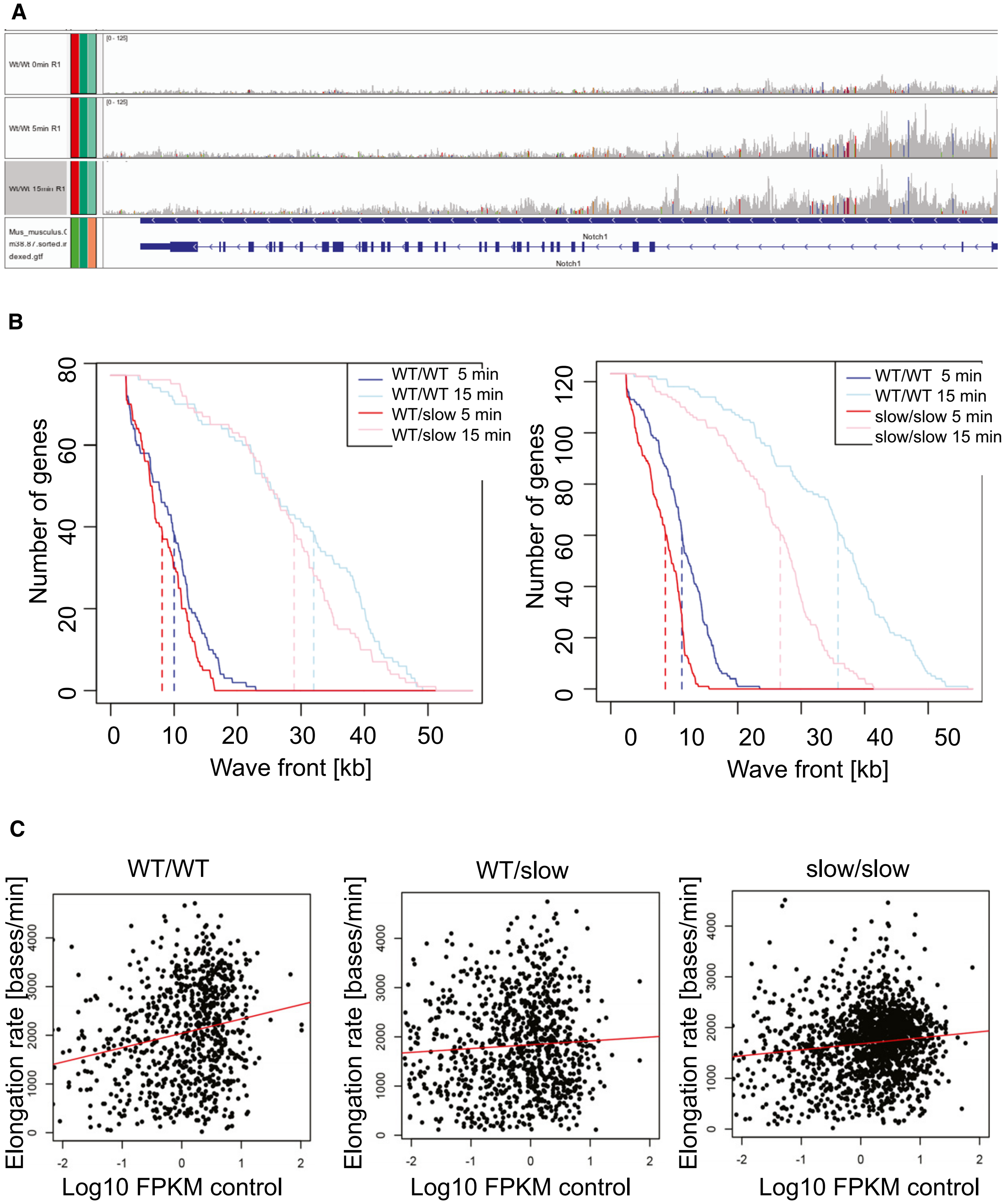

Figure EV2. Analysis of elongation rate in mouse ESCs by 4sU-DRBseq (related to Fig 3).

A Example of wave progression in the *Notch1* gene (reverse strand) following release from DRB block at 0, 5, and 15 min.

B Position of the 4sU-DRB-seq transcription wave-front for common genes over time. Dashed lines indicate median wave-front positions.

C Correlation of elongation rate measured in WT/WT, WT/slow, and slow/slow ESCs with mean expression. Correlation coefficients are 0.047, 0.003, and 0.015 (P = 2.1e-10, 0.036, and 9.4e-8), respectively.

© 2019 The Authors

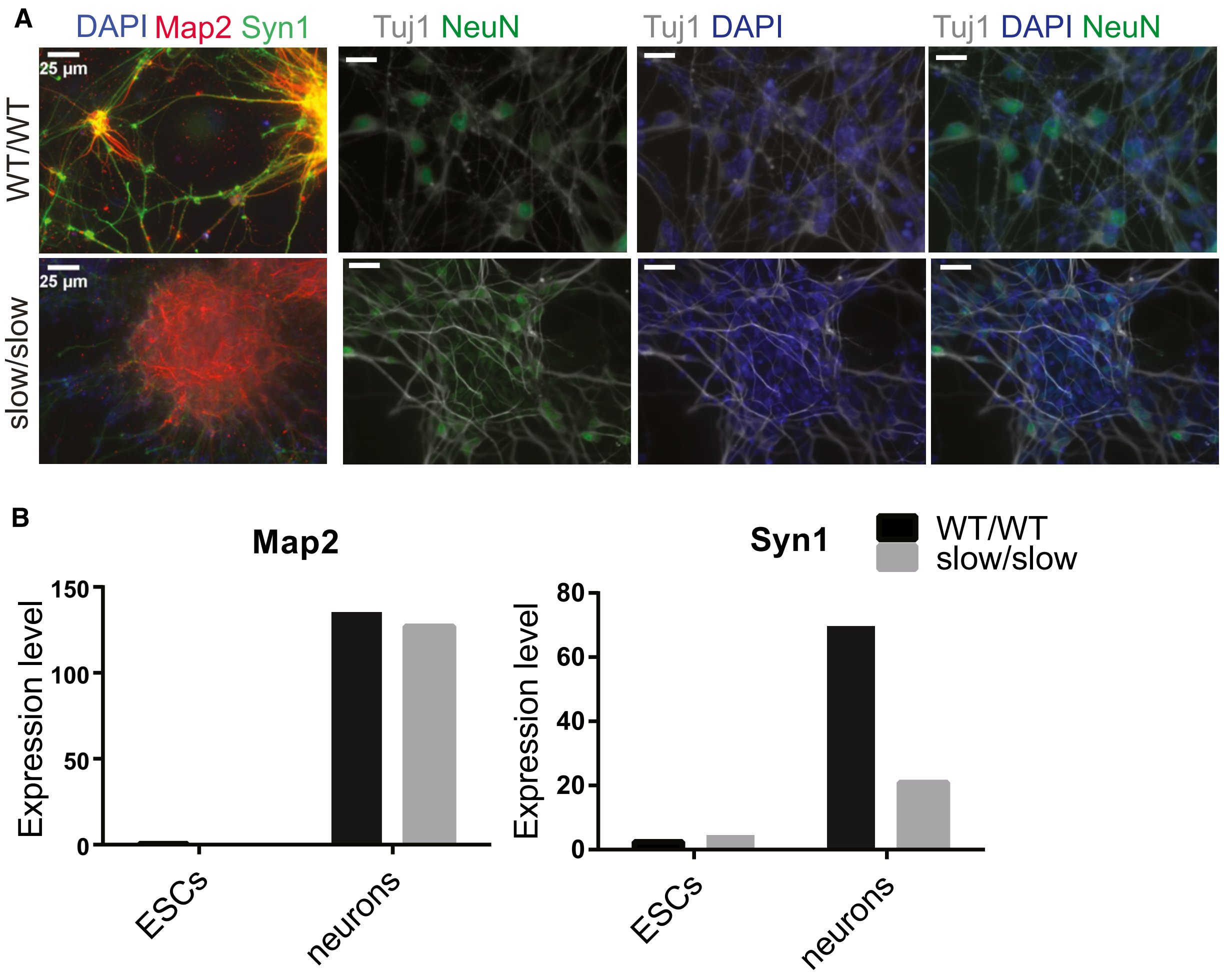

Figure EV3. Characterization of WT and slow ESCs differentiated to neurons (related to Fig 4).

A Immunofluorescence staining for neuronal markers Map2, Syn1, and NeuN in neurons cultured on poly-ornithine-/laminin-coated plates.
B Expression of neuronal markers in neurons cultured on poly-ornithine-/laminin-coated plates from RNA-seq analysis, $n = 3$.

 © 2019 The Authors

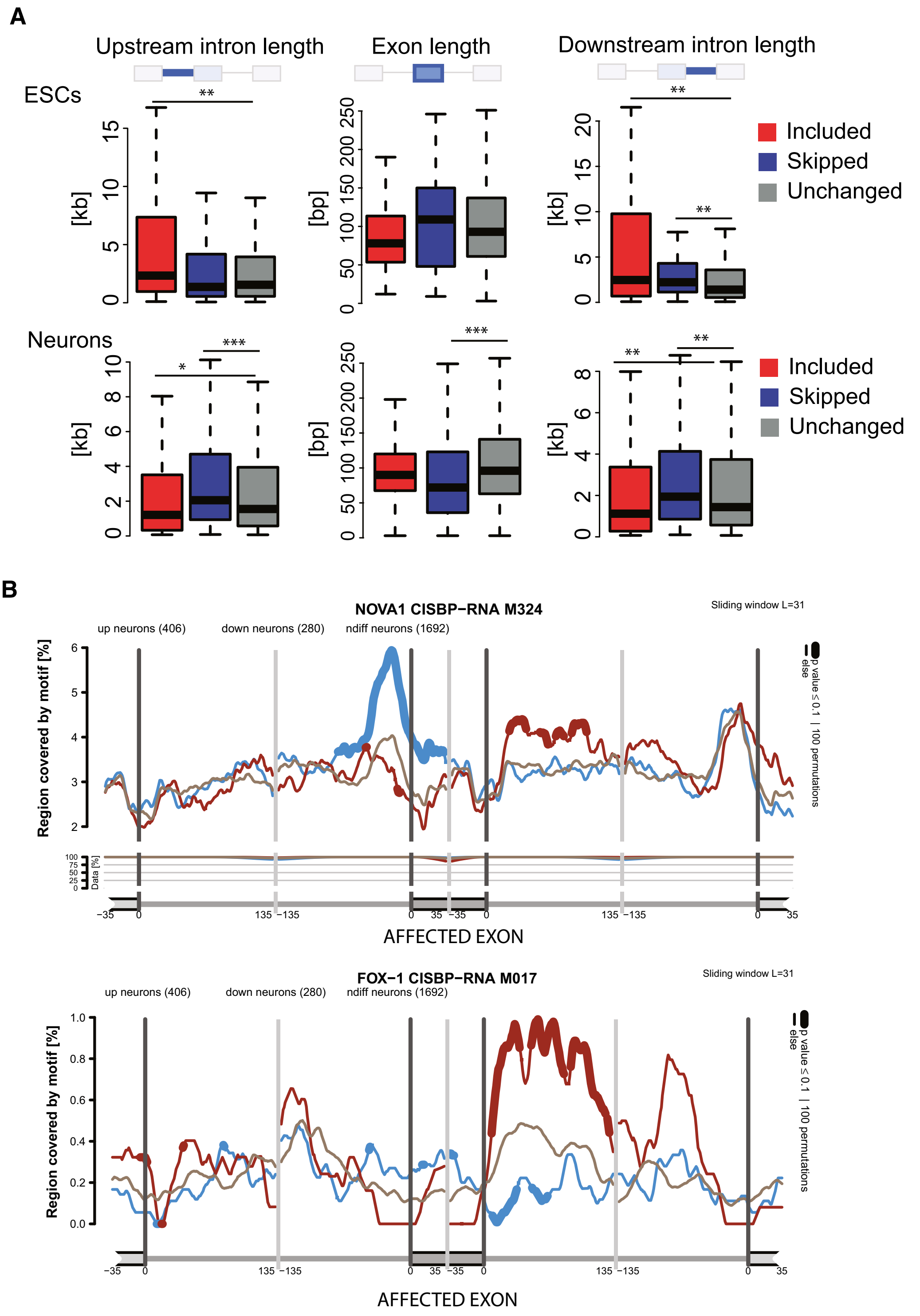

Figure EV4. Characterization of mRNAs differentially spliced in slow/slow cells (Related to Fig 5).

A Length of alternative exons and flanking introns in ESCs and neurons. Boxes delimit the first and third quartiles. The horizontal line represents the data medians. Whiskers are drawn down to the 5th and the 95th percentiles. Mann–Whitney test, $P < 0.01$ (*), 0.001 (**), 0.0001 (***).

B Example of RNA maps produced using matt for Nova1 and Fox for exons more included (blue) or less included (red) or unchanged (gray) in neurons, bold line represents $P < 0.1$. These maps reveal increased binding of these factors downstream of the regulated exon.

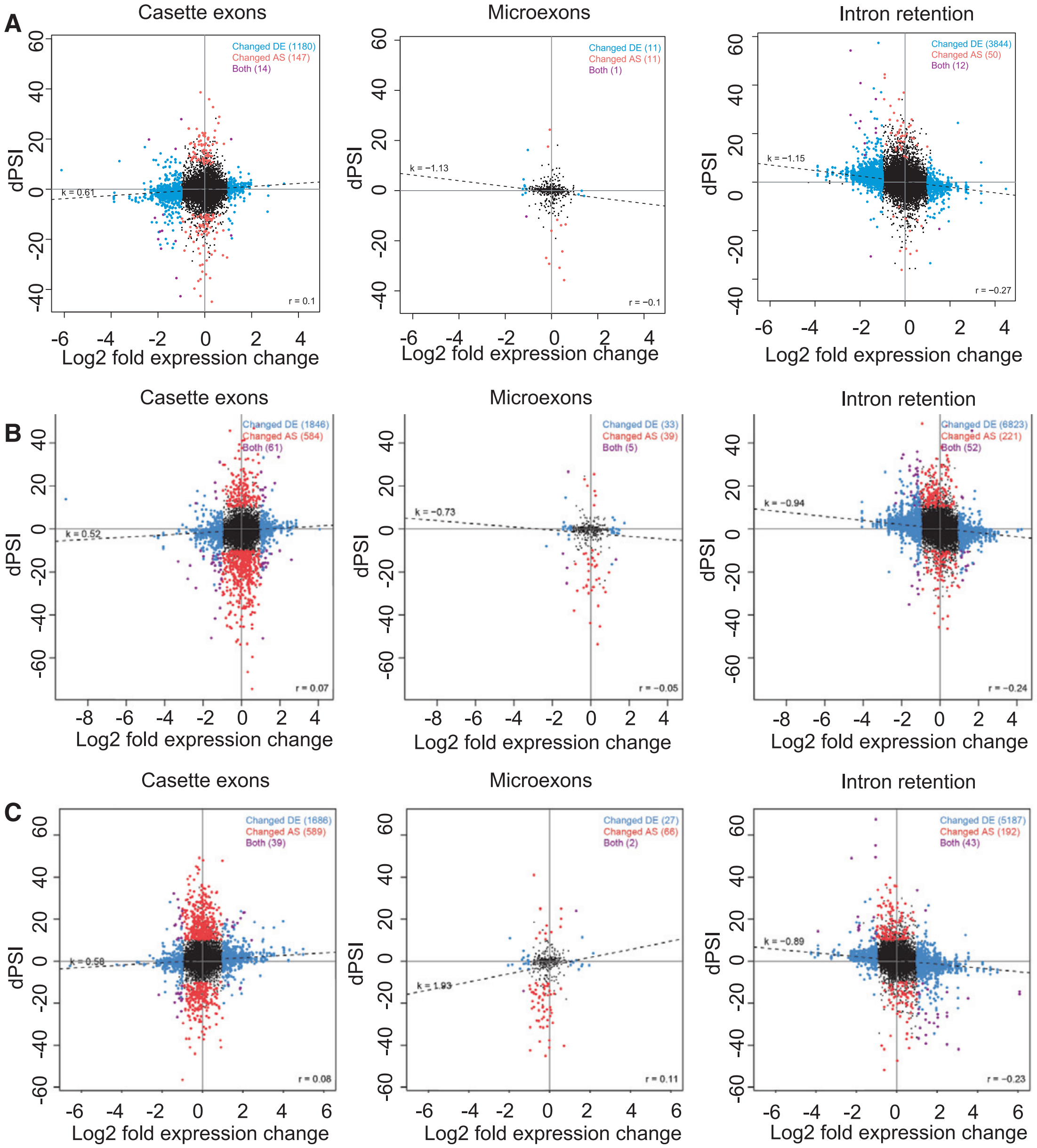

Figure EV5. Correlation of gene expression and AS changes between WT and slow/slow cells (related to Fig 5).

A–C Correlation of changes in gene expression and AS (cassette exons, microexons and intron retention events) in slow/slow cells, as compared to WT cells, in (A) ESCs, (B) NPCs and (C) neurons. The *y*-axis shows dPSI, which represents the difference in percent splicing inclusion between slow/slow and WT cells, while the *x*-axis represents a log2-fold change in expression between slow/slow and WT cells. Blue dots are for genes that change in expression (Changed DE), red dots are for differentially spliced pre-mRNAs (Changed AS), and purple dots represent both differentially expressed and spliced genes. The relation between these two variables is represented by the regression line correlation coefficients r^2.

 © 2019 The Authors
