# Supplementary material for: A slow transcription rate causes embryonic lethality and perturbs kinetic coupling of neuronal genes
Source: EMBO J. 2019 Apr 15;38(9):e101244. doi: 10.15252/embj.2018101244 (PMC6484407; doi:10.15252/embj.2018101244)
Supplement: Supplementary file 8 — Source Data for Figure 3 [file EMBJ-38-e101244-s007.zip › Source_Data_Fig3_slow.pdf]

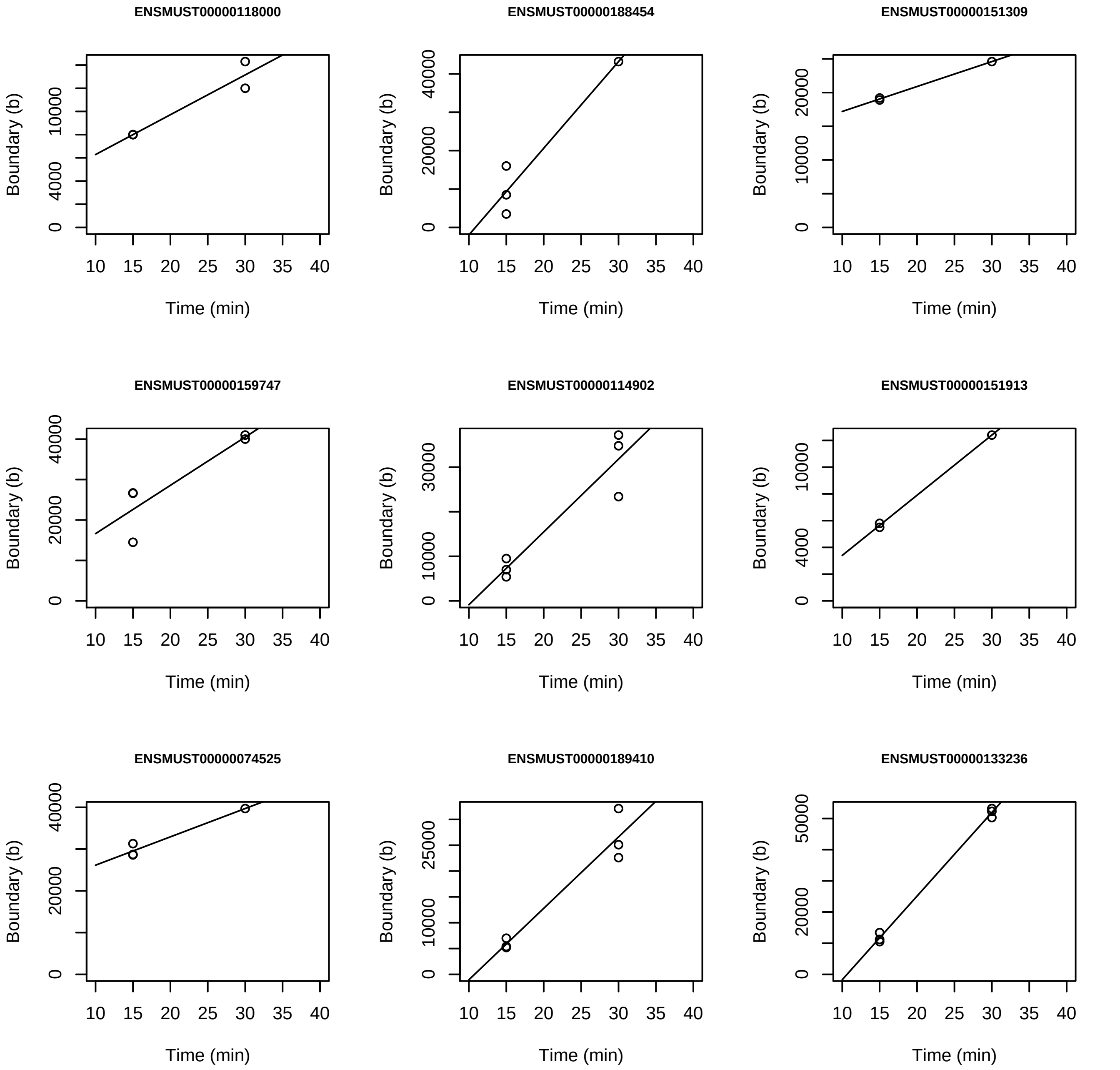
ENSMUST00000118000
ENSMUST00000188454
ENSMUST00000151309
ENSMUST00000159747
ENSMUST00000114902
ENSMUST00000151913
ENSMUST00000074525
ENSMUST00000189410
ENSMUST00000133236
Boundary (b)
Time (min)
10 15 20 25 30 35 40
0
4000
10000
20000
30000
40000
25000
50000

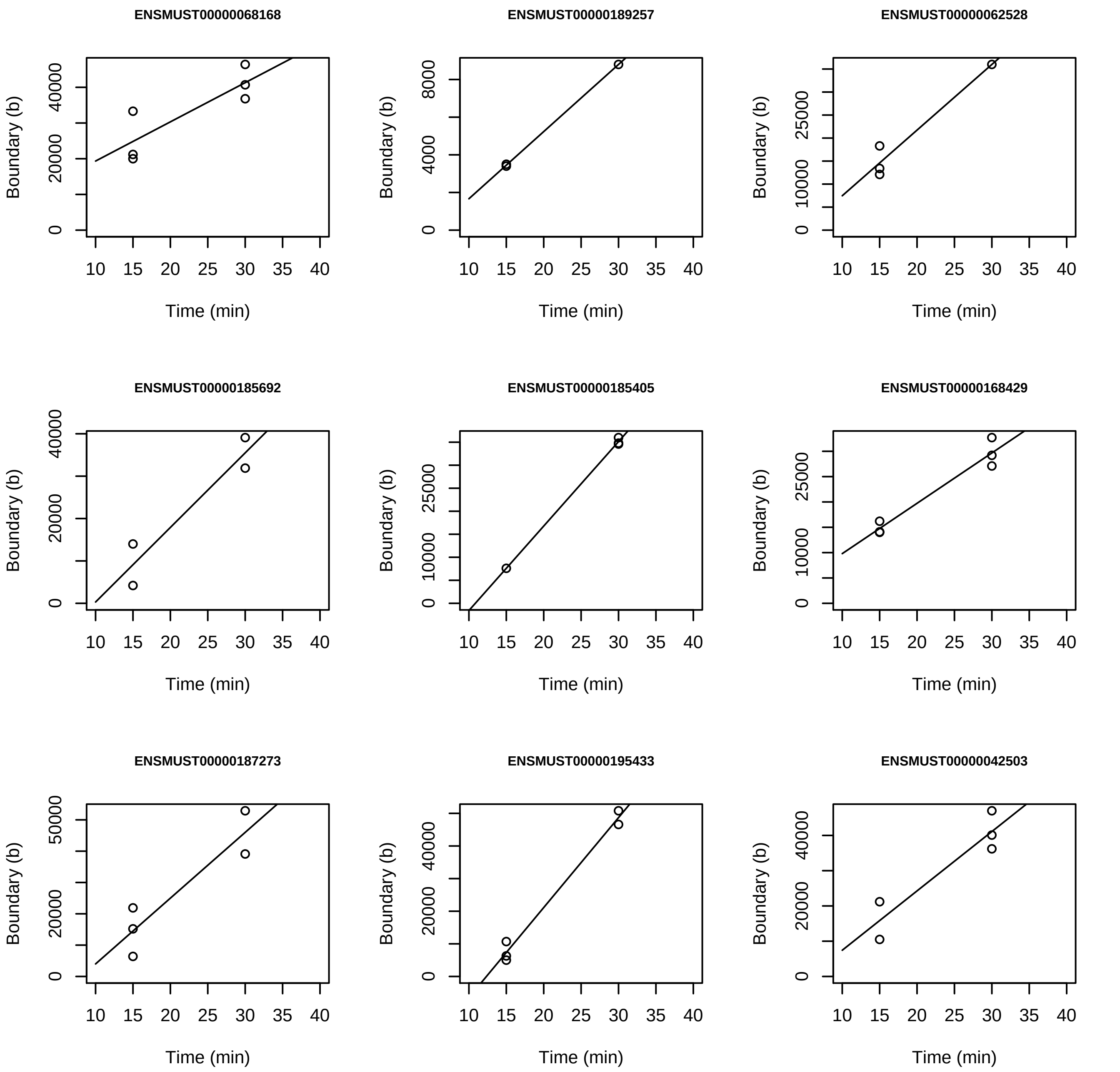
ENSMUST00000068168
ENSMUST00000189257
ENSMUST00000062528
ENSMUST00000185692
ENSMUST00000185405
ENSMUST00000168429
ENSMUST00000187273
ENSMUST00000195433
ENSMUST00000042503
Boundary (b)
Time (min)

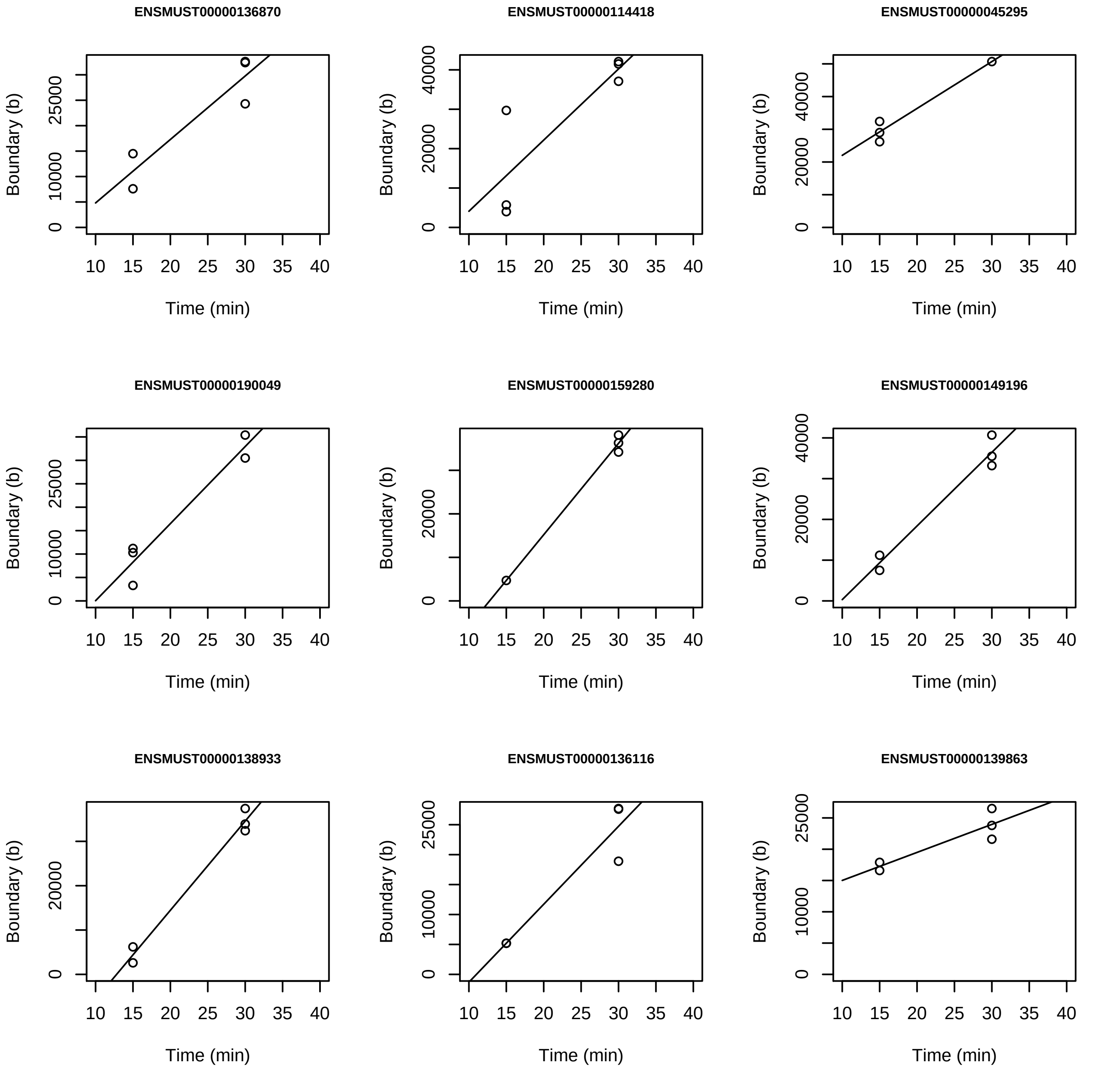
ENSMUST00000136870
ENSMUST00000114418
ENSMUST00000045295
ENSMUST00000190049
ENSMUST00000159280
ENSMUST00000149196
ENSMUST00000138933
ENSMUST00000136116
ENSMUST00000139863
Boundary (b)
Time (min)
10 15 20 25 30 35 40
0 10000 25000
0 20000 40000

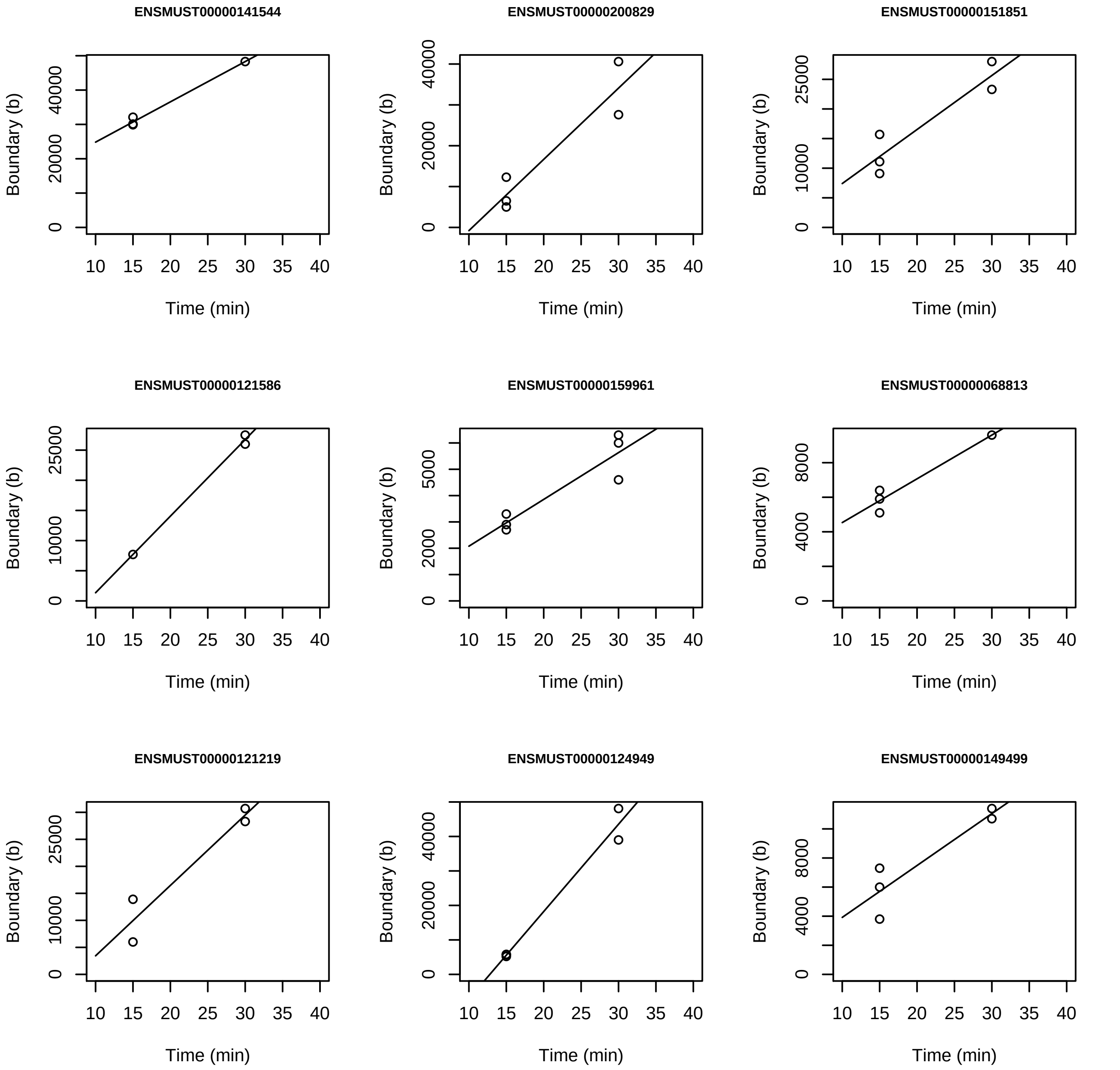
ENSMUST00000141544
ENSMUST00000200829
ENSMUST00000151851
ENSMUST00000121586
ENSMUST00000159961
ENSMUST00000068813
ENSMUST00000121219
ENSMUST00000124949
ENSMUST00000149499
Boundary (b)
Time (min)

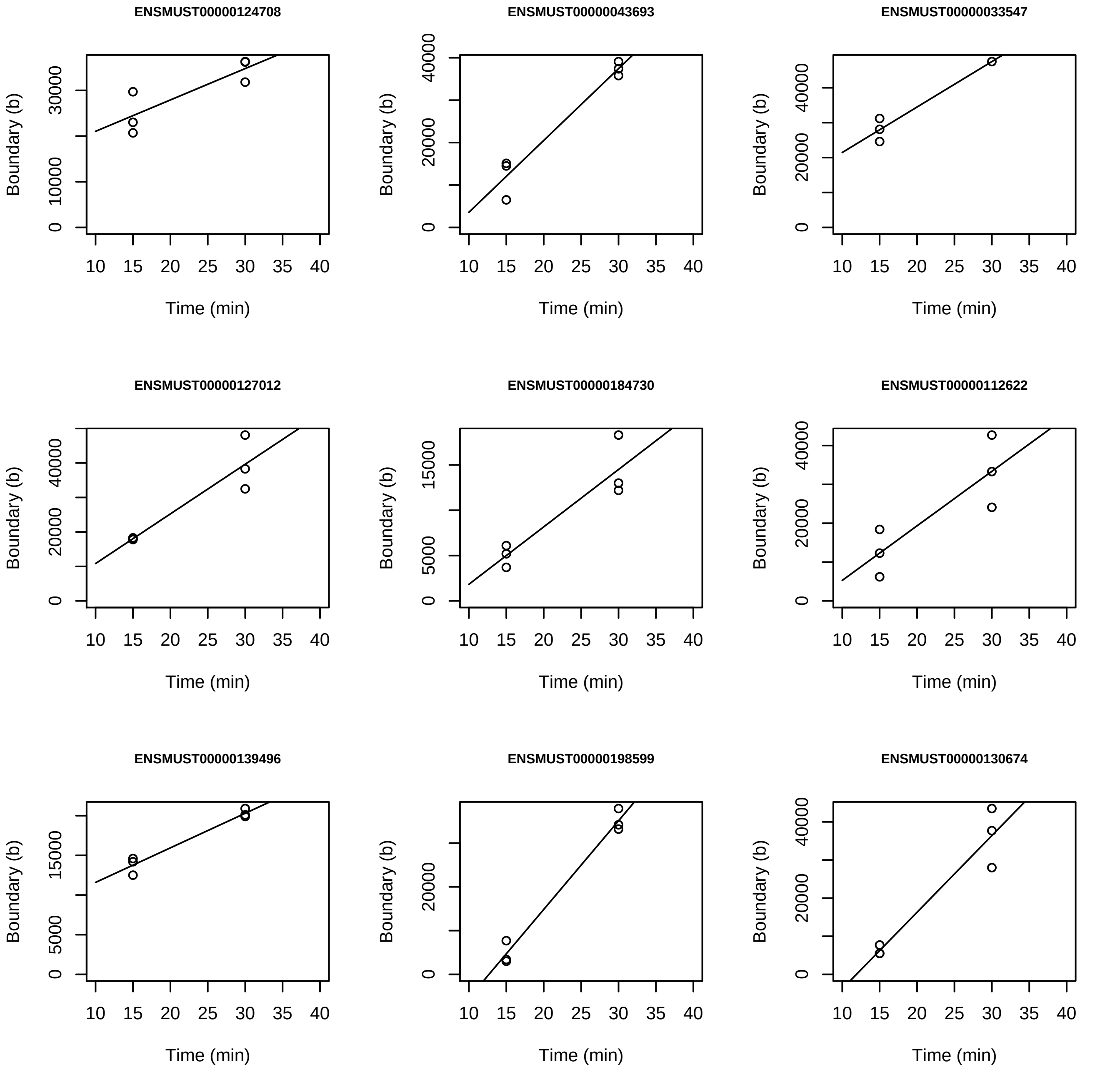

ENSMUST00000124708
ENSMUST00000043693
ENSMUST00000033547
ENSMUST00000127012
ENSMUST00000184730
ENSMUST00000112622
ENSMUST00000139496
ENSMUST00000198599
ENSMUST00000130674
Boundary (b)
Time (min)

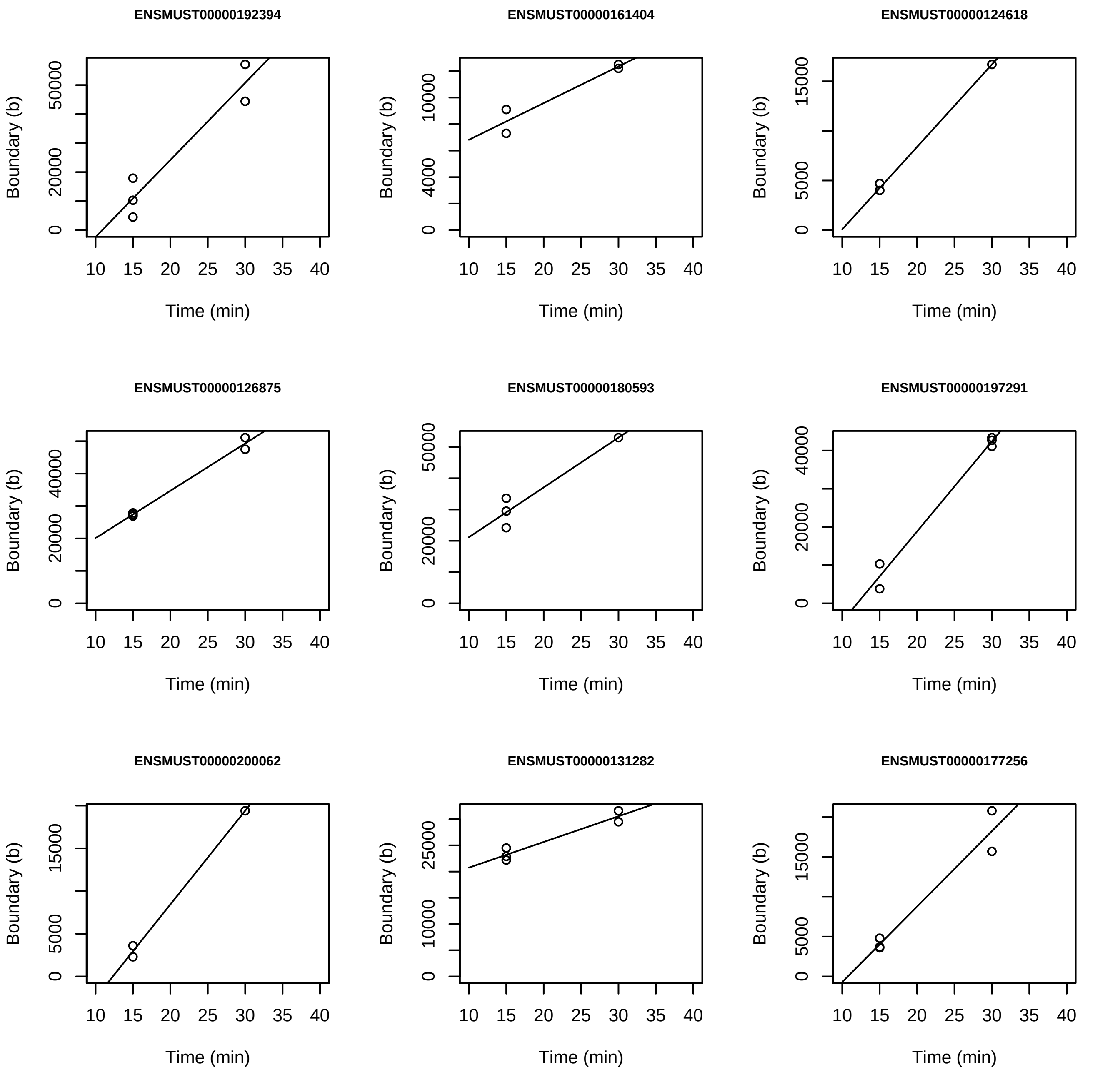
ENSMUST00000192394
ENSMUST00000161404
ENSMUST00000124618
ENSMUST00000126875
ENSMUST00000180593
ENSMUST00000197291
ENSMUST00000200062
ENSMUST00000131282
ENSMUST00000177256
Boundary (b)
Time (min)

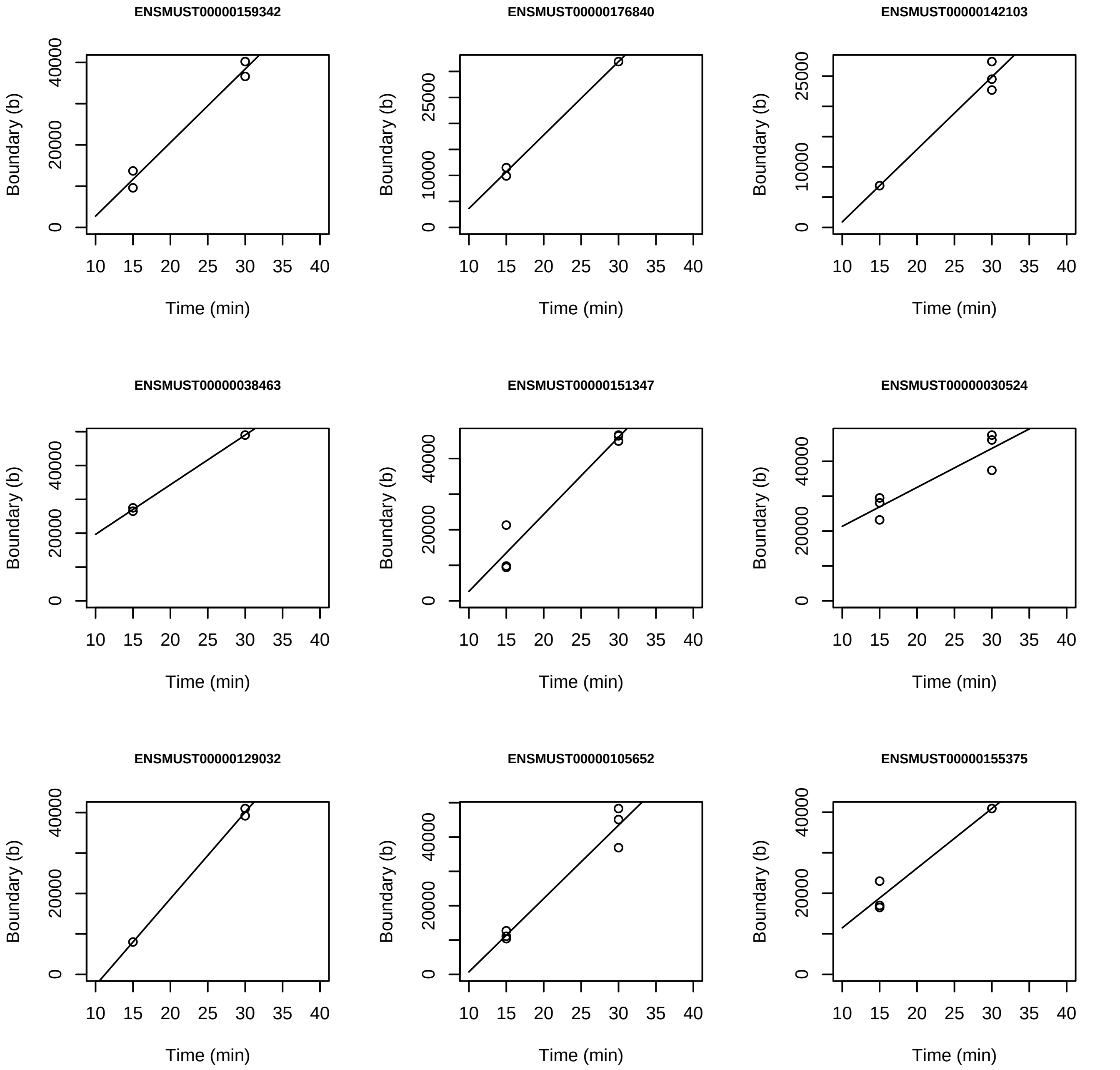

ENSMUST00000159342
ENSMUST00000176840
ENSMUST00000142103
ENSMUST00000038463
ENSMUST00000151347
ENSMUST00000030524
ENSMUST00000129032
ENSMUST00000105652
ENSMUST00000155375
Boundary (b)
Time (min)

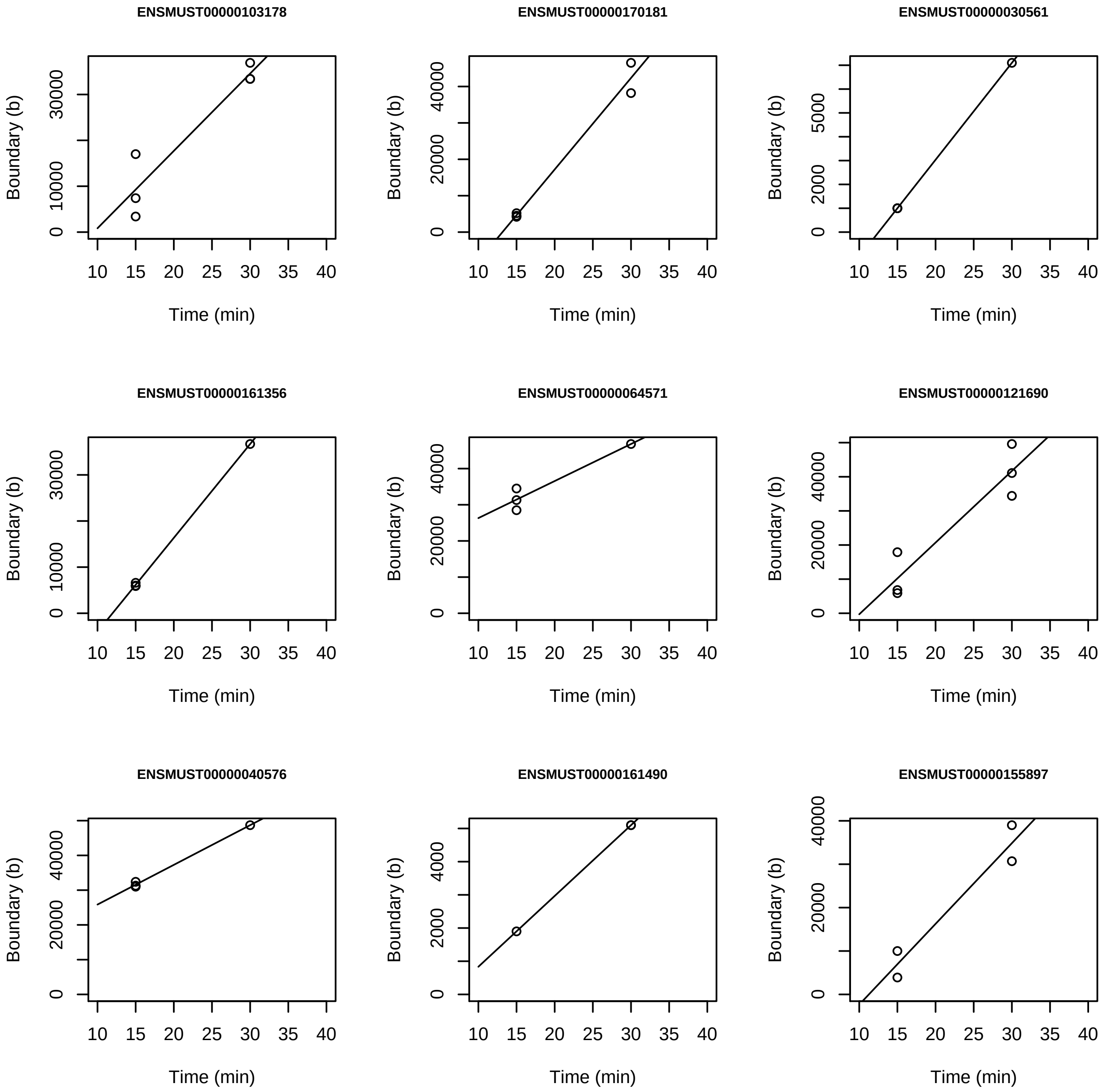
ENSMUST00000103178
ENSMUST00000170181
ENSMUST00000030561
ENSMUST00000161356
ENSMUST00000064571
ENSMUST00000121690
ENSMUST00000040576
ENSMUST00000161490
ENSMUST00000155897
Boundary (b)
Time (min)

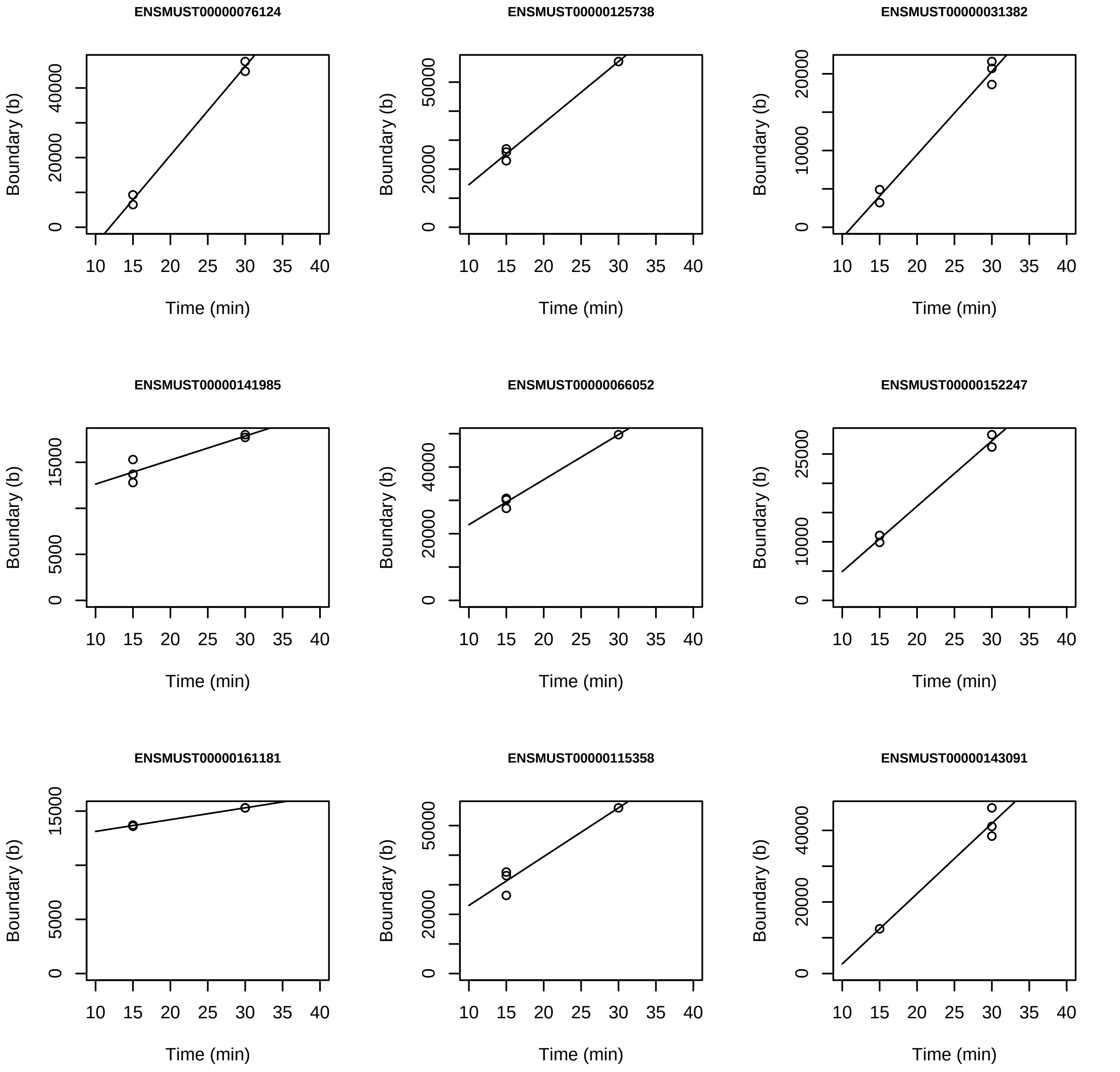
ENSMUST00000076124
ENSMUST00000125738
ENSMUST00000031382
ENSMUST00000141985
ENSMUST00000066052
ENSMUST00000152247
ENSMUST00000161181
ENSMUST00000115358
ENSMUST00000143091
Boundary (b)
Time (min)
0
5000
10000
15000
20000
25000
40000
50000
10
15
20
25
30
35
40

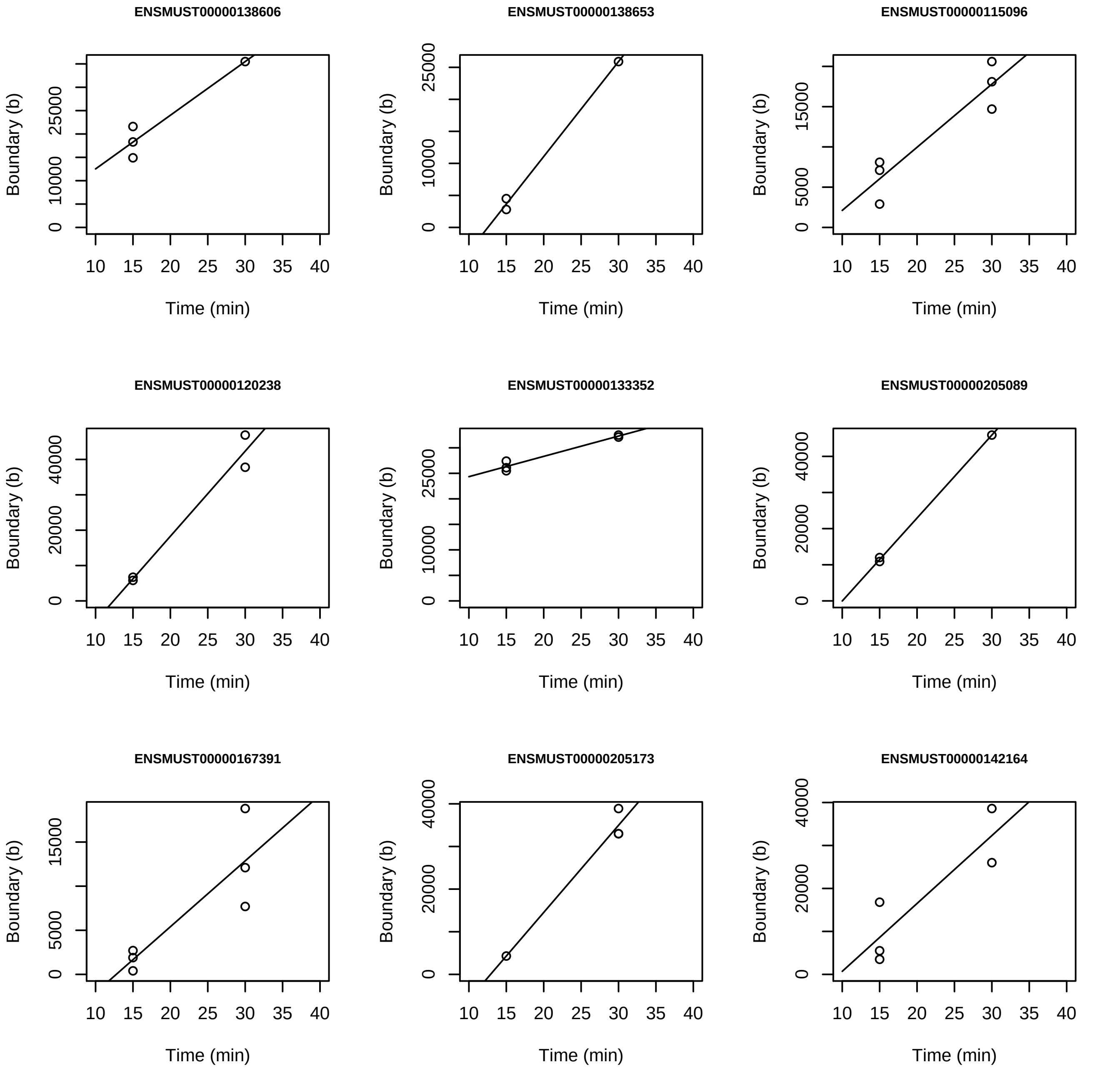

ENSMUST00000138606
ENSMUST00000138653
ENSMUST00000115096
ENSMUST00000120238
ENSMUST00000133352
ENSMUST00000205089
ENSMUST00000167391
ENSMUST00000205173
ENSMUST00000142164
Boundary (b)
Time (min)

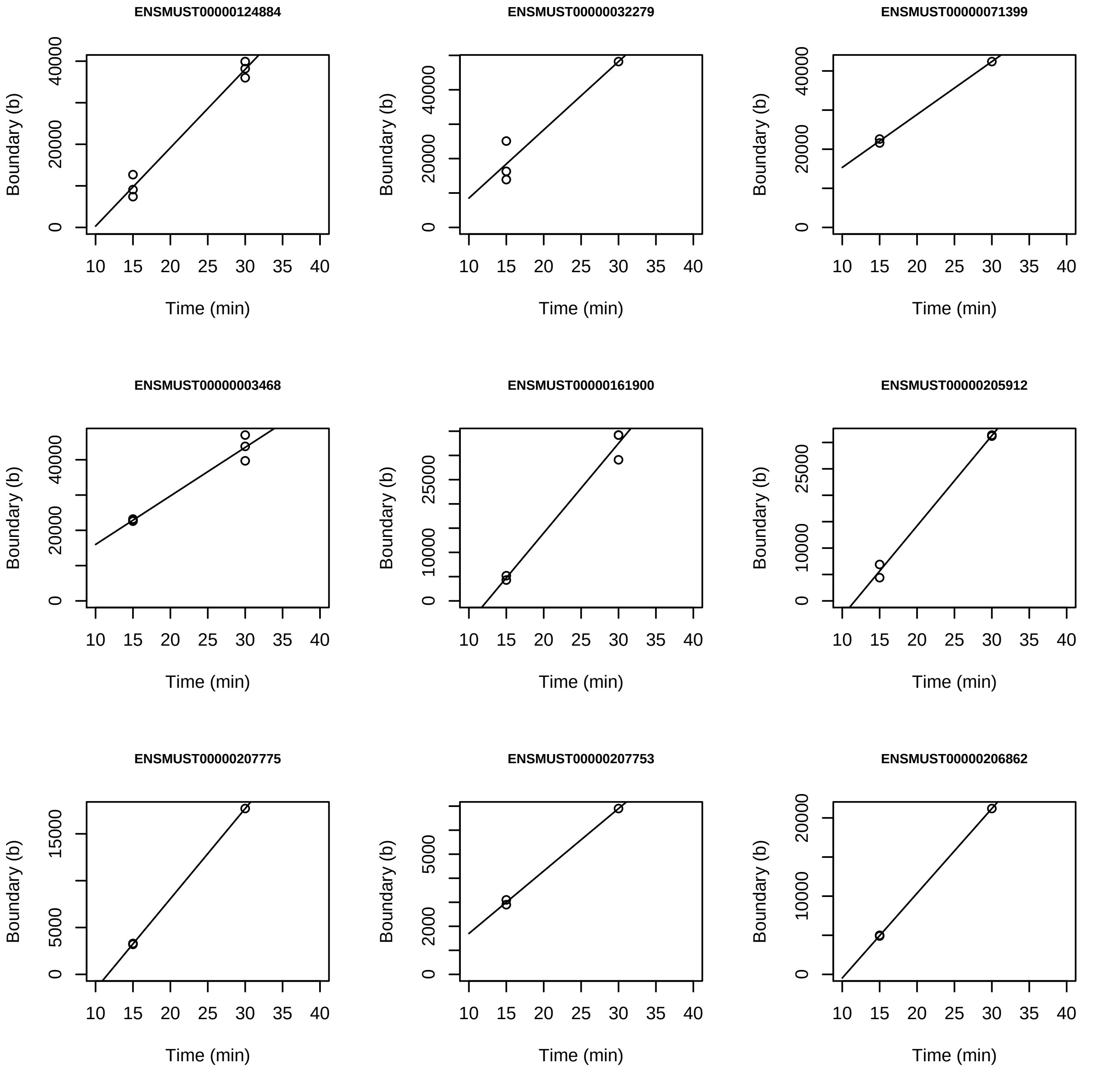

ENSMUST00000124884
ENSMUST00000032279
ENSMUST00000071399
ENSMUST00000003468
ENSMUST00000161900
ENSMUST00000205912
ENSMUST00000207775
ENSMUST00000207753
ENSMUST00000206862
Boundary (b)
Time (min)

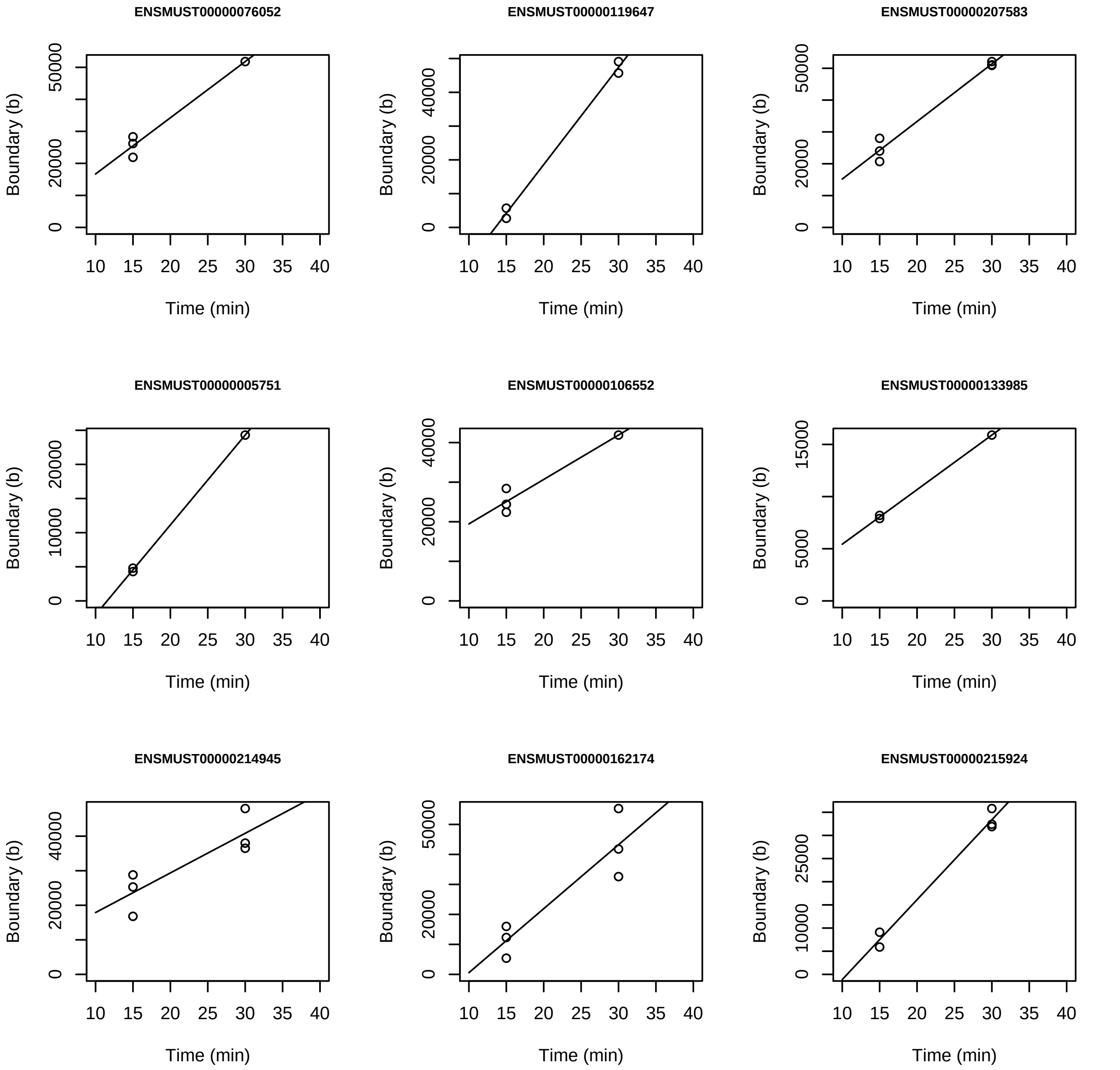
ENSMUST00000076052
ENSMUST00000119647
ENSMUST00000207583
ENSMUST00000005751
ENSMUST00000106552
ENSMUST00000133985
ENSMUST00000214945
ENSMUST00000162174
ENSMUST00000215924
Boundary (b)
Time (min)
0
5000
10000
15000
20000
25000
40000
50000
10
15
20
25
30
35
40

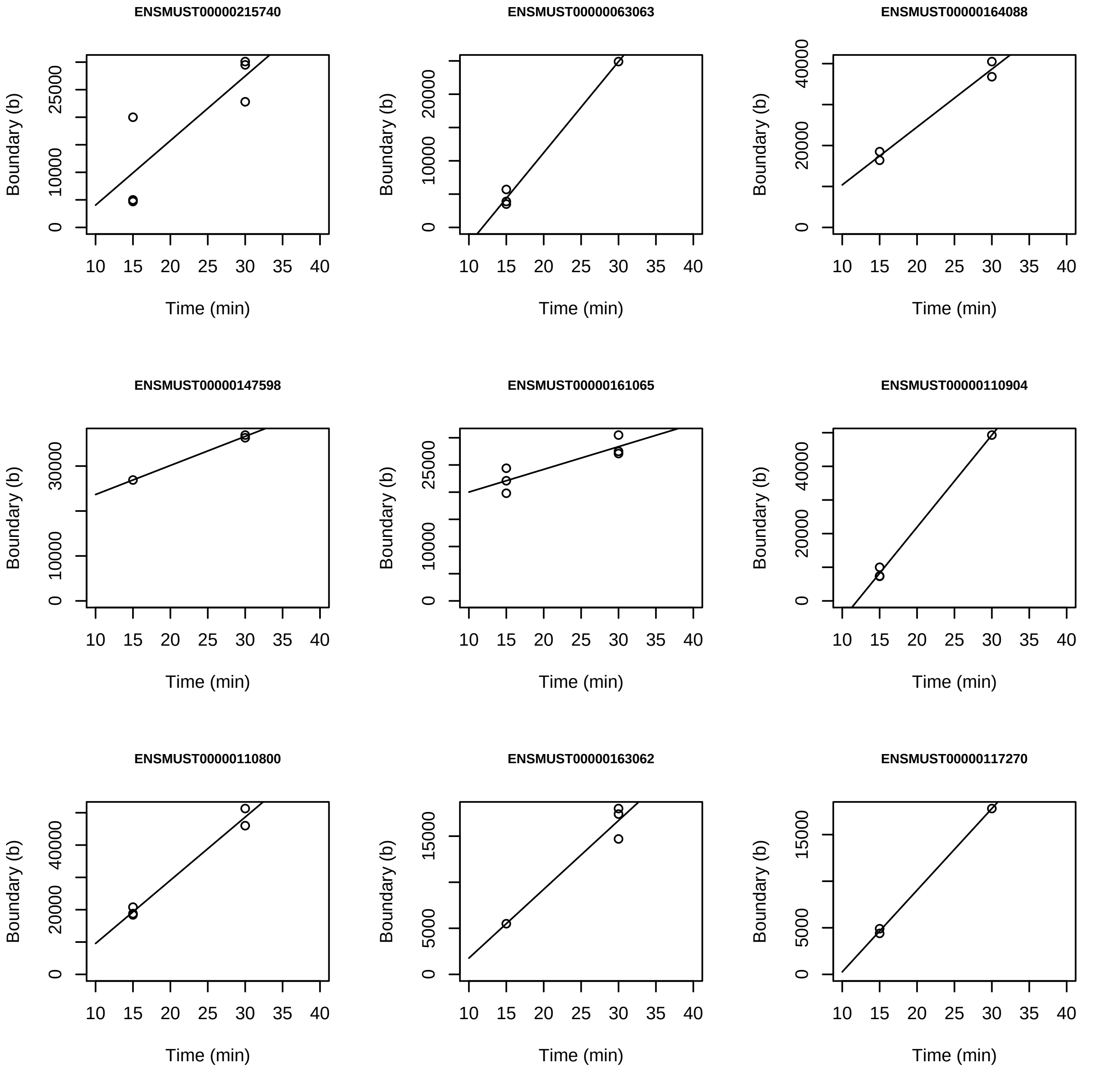
ENSMUST00000215740
ENSMUST00000063063
ENSMUST00000164088
ENSMUST00000147598
ENSMUST00000161065
ENSMUST00000110904
ENSMUST00000110800
ENSMUST00000163062
ENSMUST00000117270
Boundary (b)
Time (min)
10 15 20 25 30 35 40
0 10000 25000
0 10000 20000
0 20000 40000
0 10000 30000
0 10000 25000
0 20000 40000
0 20000 40000
0 5000 15000
0 5000 15000

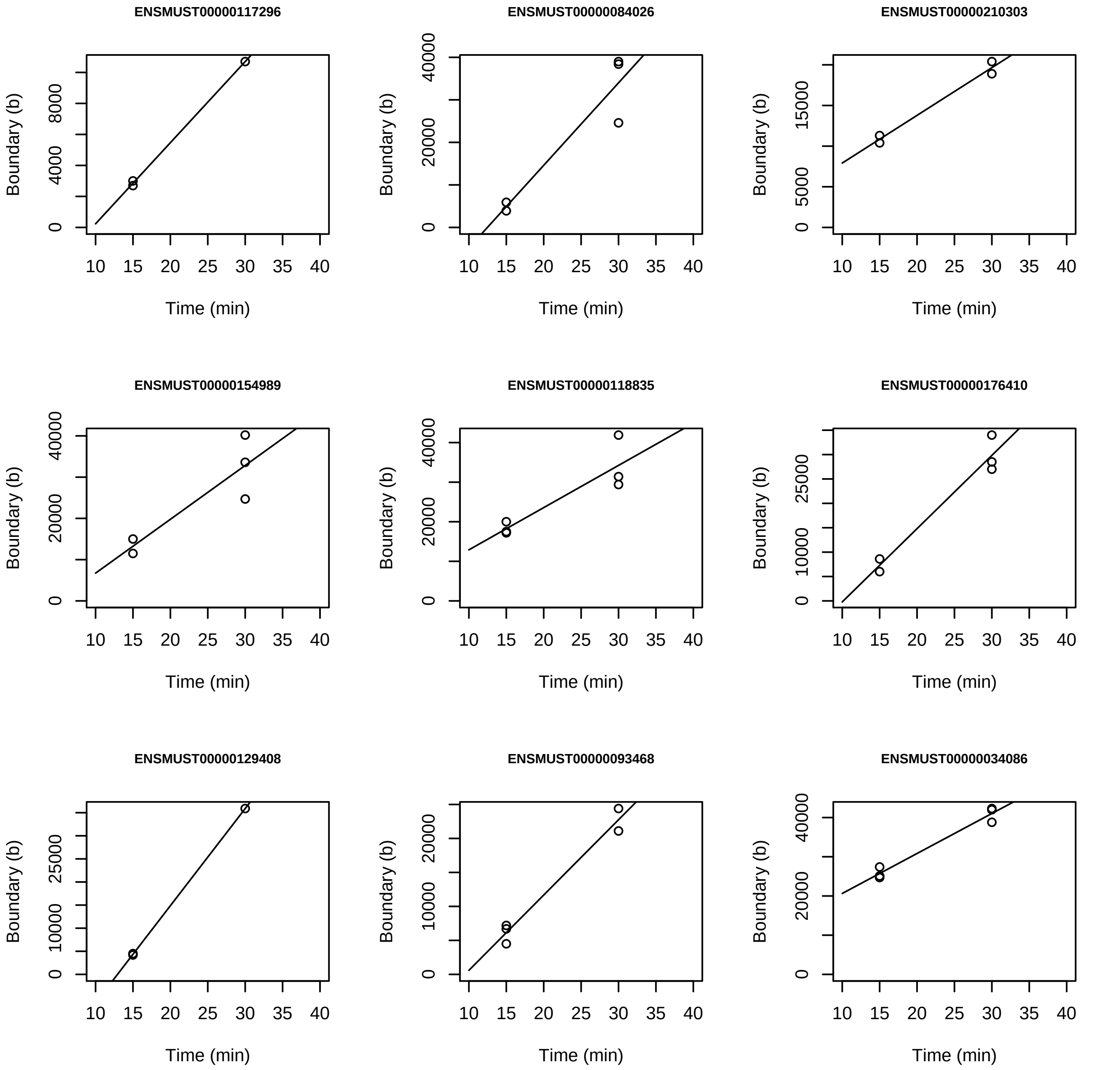
ENSMUST00000117296
ENSMUST00000084026
ENSMUST00000210303
ENSMUST00000154989
ENSMUST00000118835
ENSMUST00000176410
ENSMUST00000129408
ENSMUST00000093468
ENSMUST00000034086
Boundary (b)
Time (min)
10 15 20 25 30 35 40

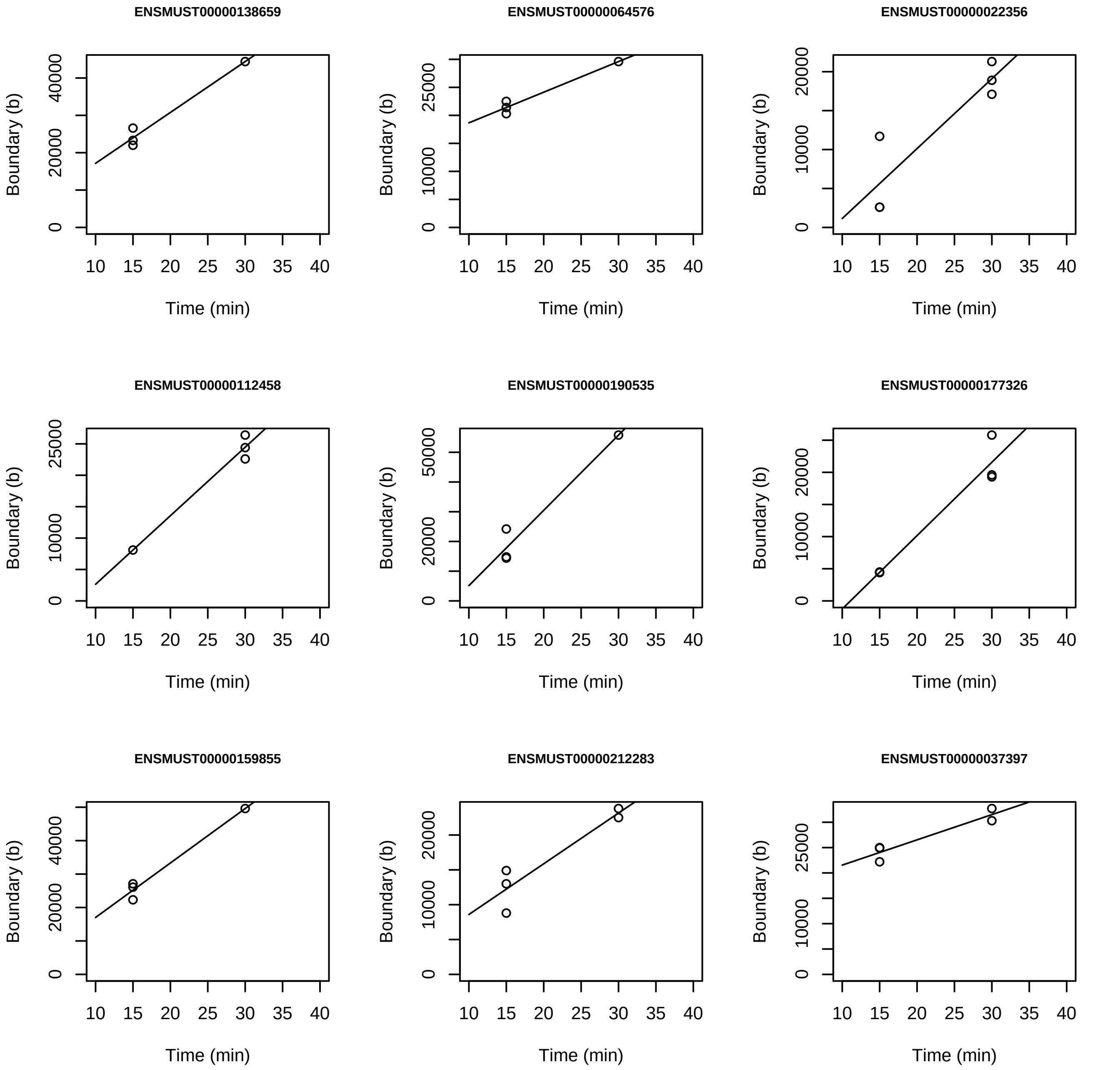
ENSMUST00000138659
Boundary (b)
0
20000
40000
10
15
20
25
30
35
40
Time (min)
ENSMUST00000064576
Boundary (b)
0
10000
25000
Time (min)
ENSMUST00000022356
Boundary (b)
0
10000
20000
Time (min)
ENSMUST00000112458
Boundary (b)
0
10000
25000
Time (min)
ENSMUST00000190535
Boundary (b)
0
20000
50000
Time (min)
ENSMUST00000177326
Boundary (b)
0
10000
20000
Time (min)
ENSMUST00000159855
Boundary (b)
0
20000
40000
Time (min)
ENSMUST00000212283
Boundary (b)
0
10000
20000
Time (min)
ENSMUST00000037397
Boundary (b)
0
10000
25000
Time (min)

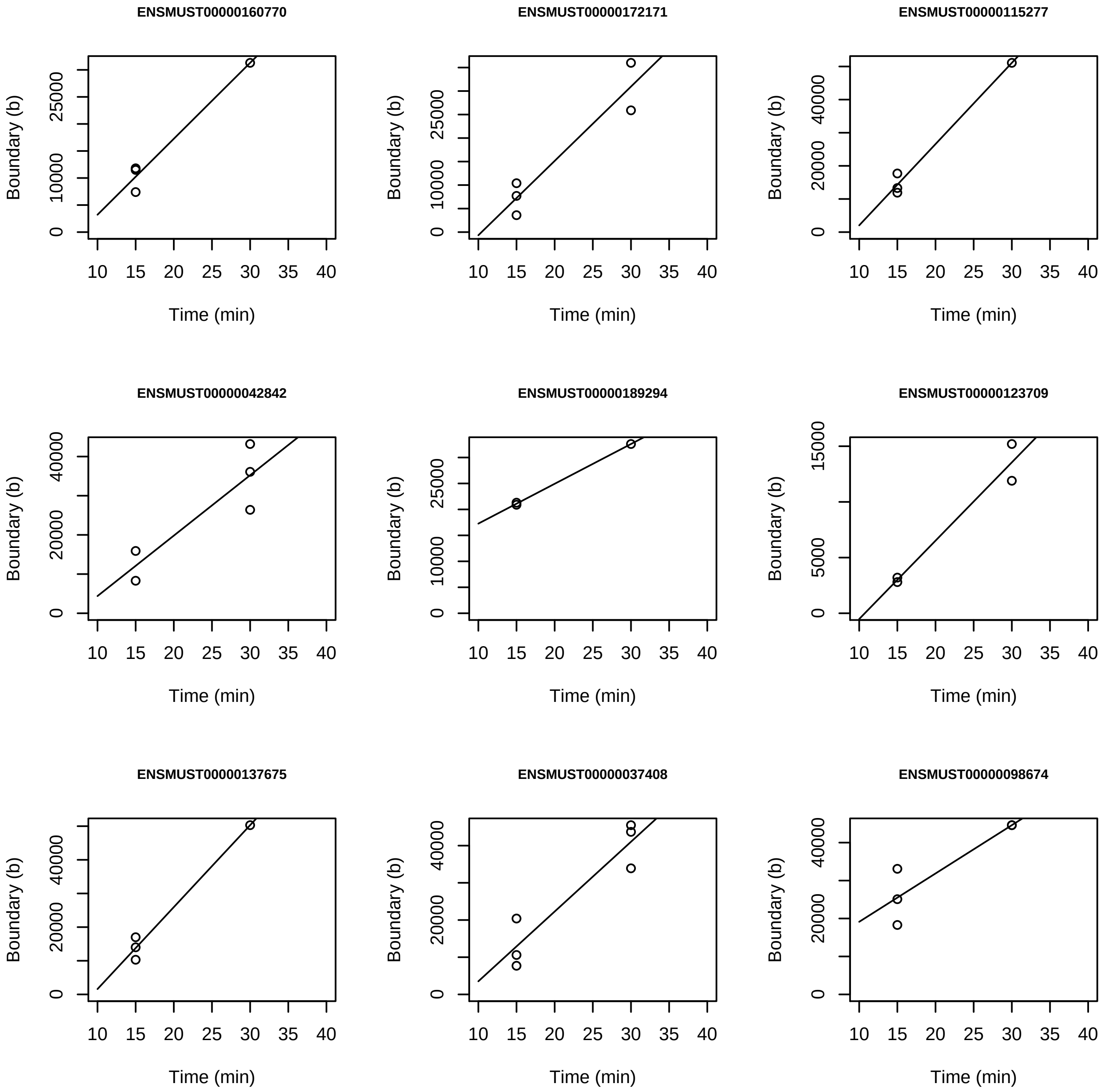

ENSMUST00000160770
ENSMUST00000172171
ENSMUST00000115277
ENSMUST00000042842
ENSMUST00000189294
ENSMUST00000123709
ENSMUST00000137675
ENSMUST00000037408
ENSMUST00000098674
Boundary (b)
Time (min)
10 15 20 25 30 35 40
0 10000 25000
0 10000 25000
0 20000 40000
0 20000 40000
0 10000 25000
0 5000 15000
0 20000 40000
0 20000 40000
0 20000 40000

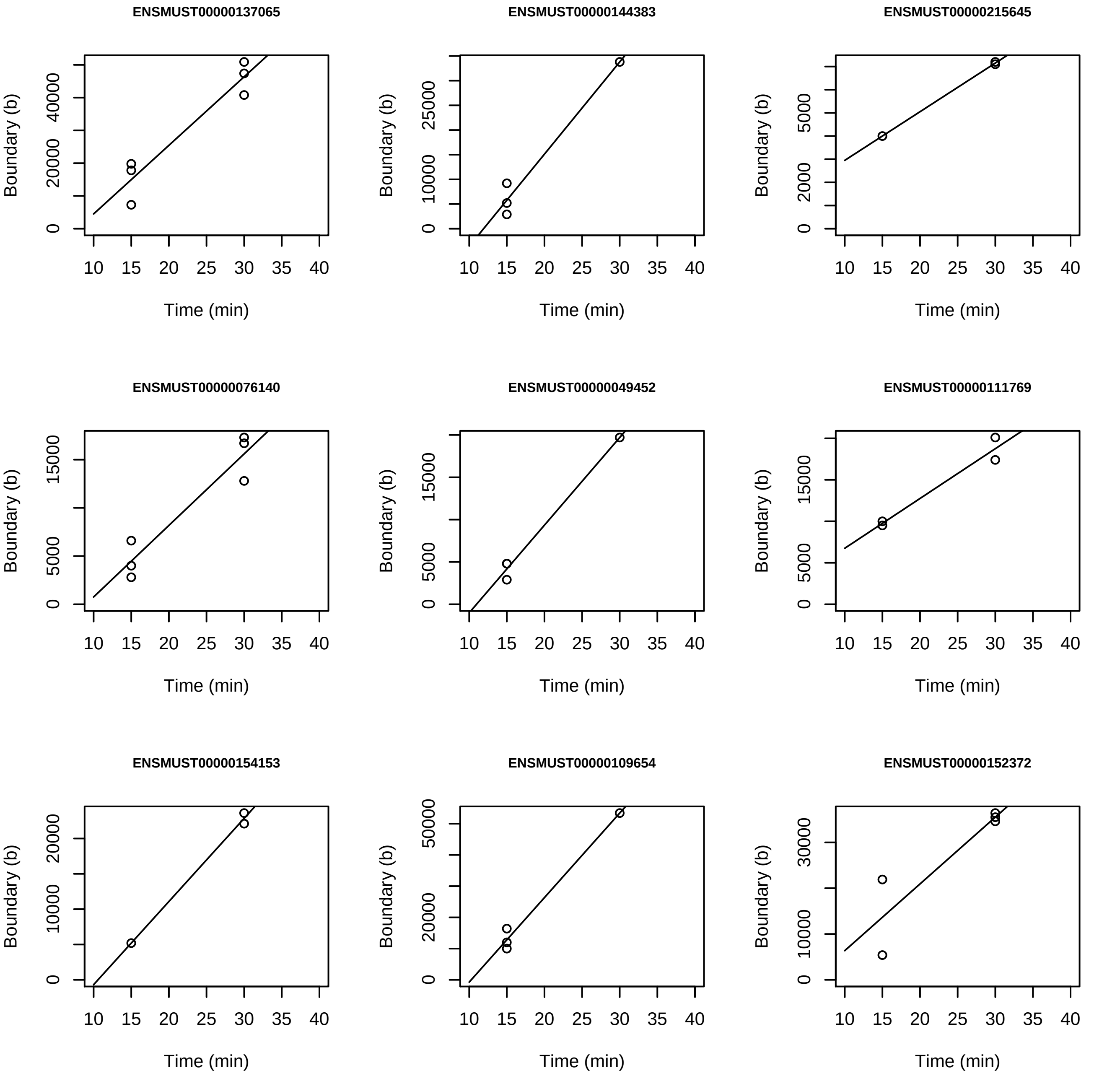
ENSMUST00000137065
ENSMUST00000144383
ENSMUST00000215645
ENSMUST00000076140
ENSMUST00000049452
ENSMUST00000111769
ENSMUST00000154153
ENSMUST00000109654
ENSMUST00000152372
Boundary (b)
Time (min)

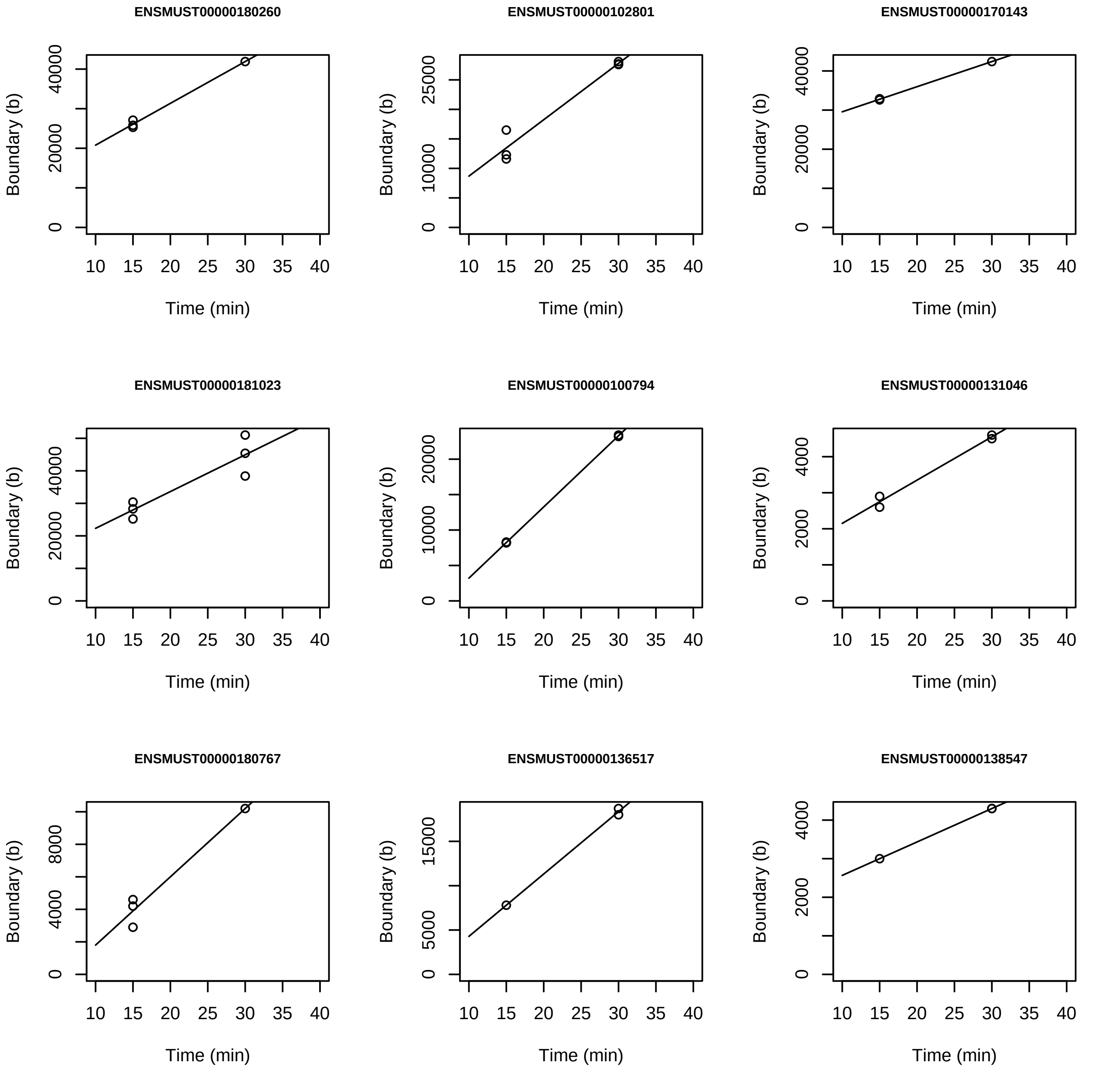
ENSMUST00000180260
ENSMUST00000102801
ENSMUST00000170143
ENSMUST00000181023
ENSMUST00000100794
ENSMUST00000131046
ENSMUST00000180767
ENSMUST00000136517
ENSMUST00000138547
Boundary (b)
Time (min)
10 15 20 25 30 35 40
0 20000 40000
0 10000 25000
0 10000 20000
0 2000 4000
0 4000 8000
0 5000 15000

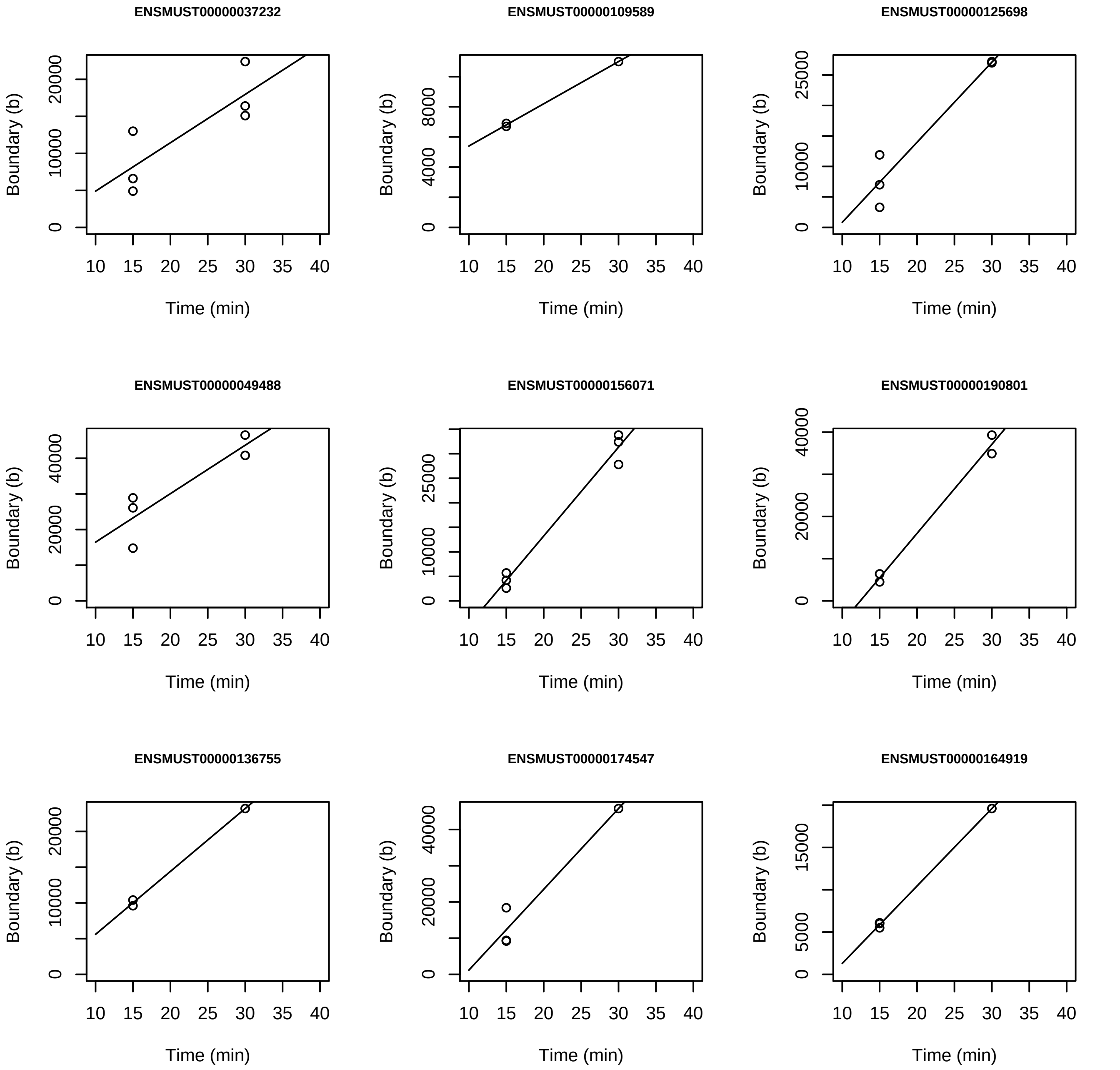
ENSMUST00000037232
ENSMUST00000109589
ENSMUST00000125698
ENSMUST00000049488
ENSMUST00000156071
ENSMUST00000190801
ENSMUST00000136755
ENSMUST00000174547
ENSMUST00000164919
Boundary (b)
Time (min)
10 15 20 25 30 35 40
0 10000 20000
0 4000 8000
0 10000 25000
0 20000 40000
0 10000 25000
0 20000 40000
0 10000 20000
0 20000 40000
0 5000 15000

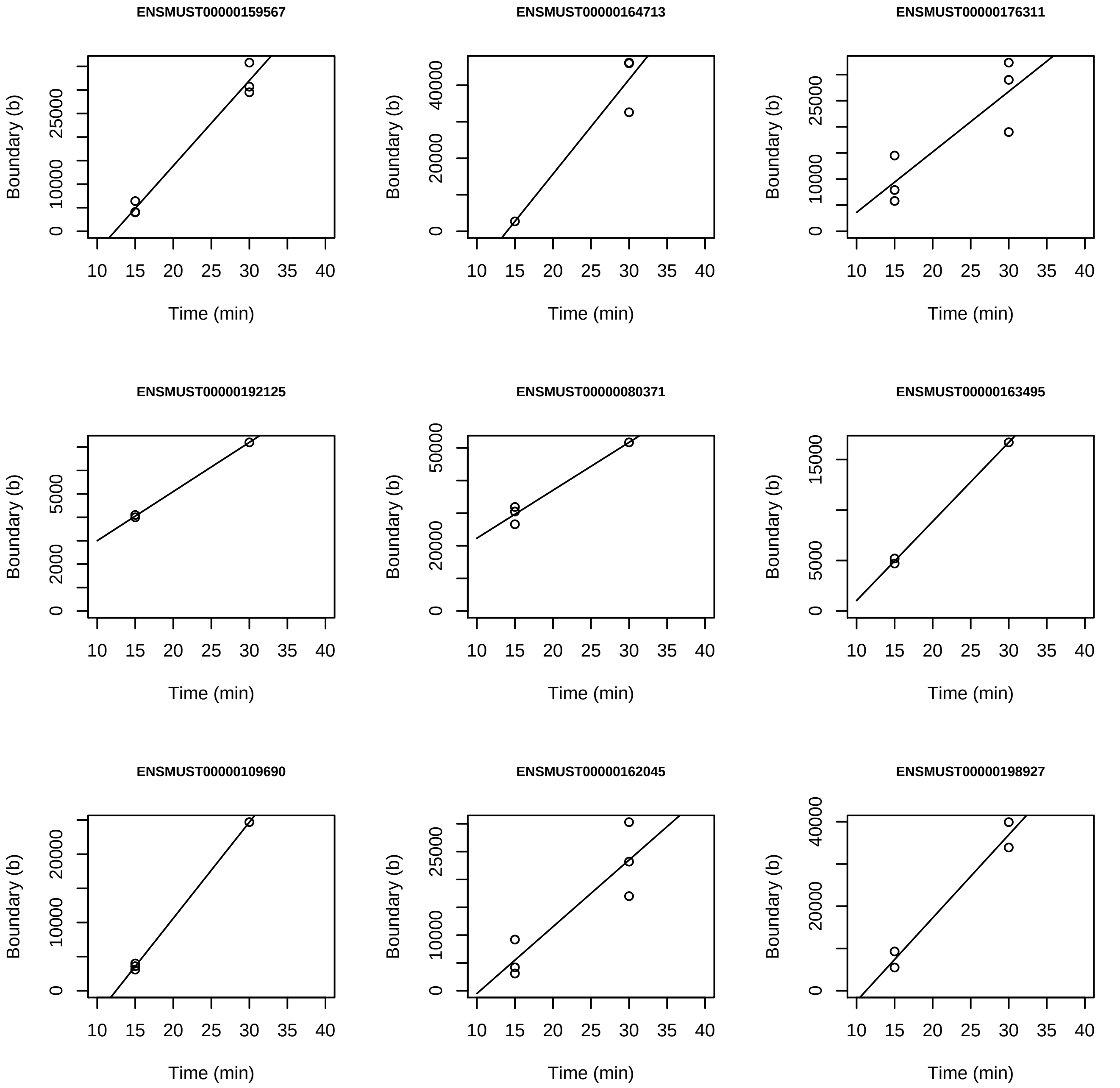
ENSMUST00000159567
ENSMUST00000164713
ENSMUST00000176311
ENSMUST00000192125
ENSMUST00000080371
ENSMUST00000163495
ENSMUST00000109690
ENSMUST00000162045
ENSMUST00000198927
Boundary (b)
Time (min)
10 15 20 25 30 35 40
0 10000 25000
0 20000 40000
0 10000 25000
0 2000 5000
0 20000 50000
0 5000 15000
0 10000 20000
0 10000 25000
0 20000 40000

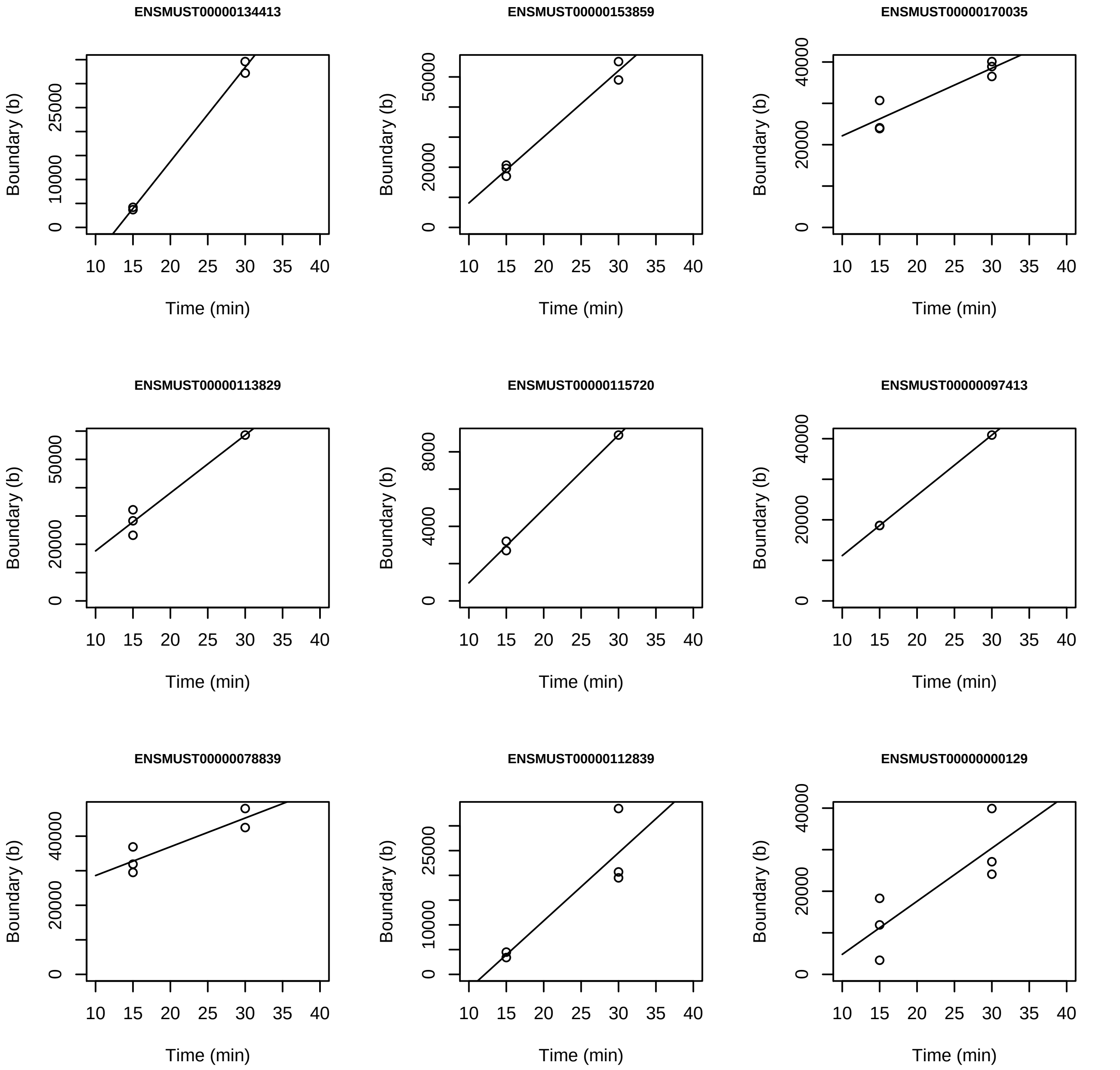
ENSMUST00000134413
Boundary (b)
0
10000
25000
Time (min)
10 15 20 25 30 35 40
ENSMUST00000153859
Boundary (b)
0
20000
50000
Time (min)
10 15 20 25 30 35 40
ENSMUST00000170035
Boundary (b)
0
20000
40000
Time (min)
10 15 20 25 30 35 40
ENSMUST00000113829
Boundary (b)
0
20000
50000
Time (min)
10 15 20 25 30 35 40
ENSMUST00000115720
Boundary (b)
0
4000
8000
Time (min)
10 15 20 25 30 35 40
ENSMUST00000097413
Boundary (b)
0
20000
40000
Time (min)
10 15 20 25 30 35 40
ENSMUST00000078839
Boundary (b)
0
20000
40000
Time (min)
10 15 20 25 30 35 40
ENSMUST00000112839
Boundary (b)
0
10000
25000
Time (min)
10 15 20 25 30 35 40
ENSMUST00000000129
Boundary (b)
0
20000
40000
Time (min)
10 15 20 25 30 35 40

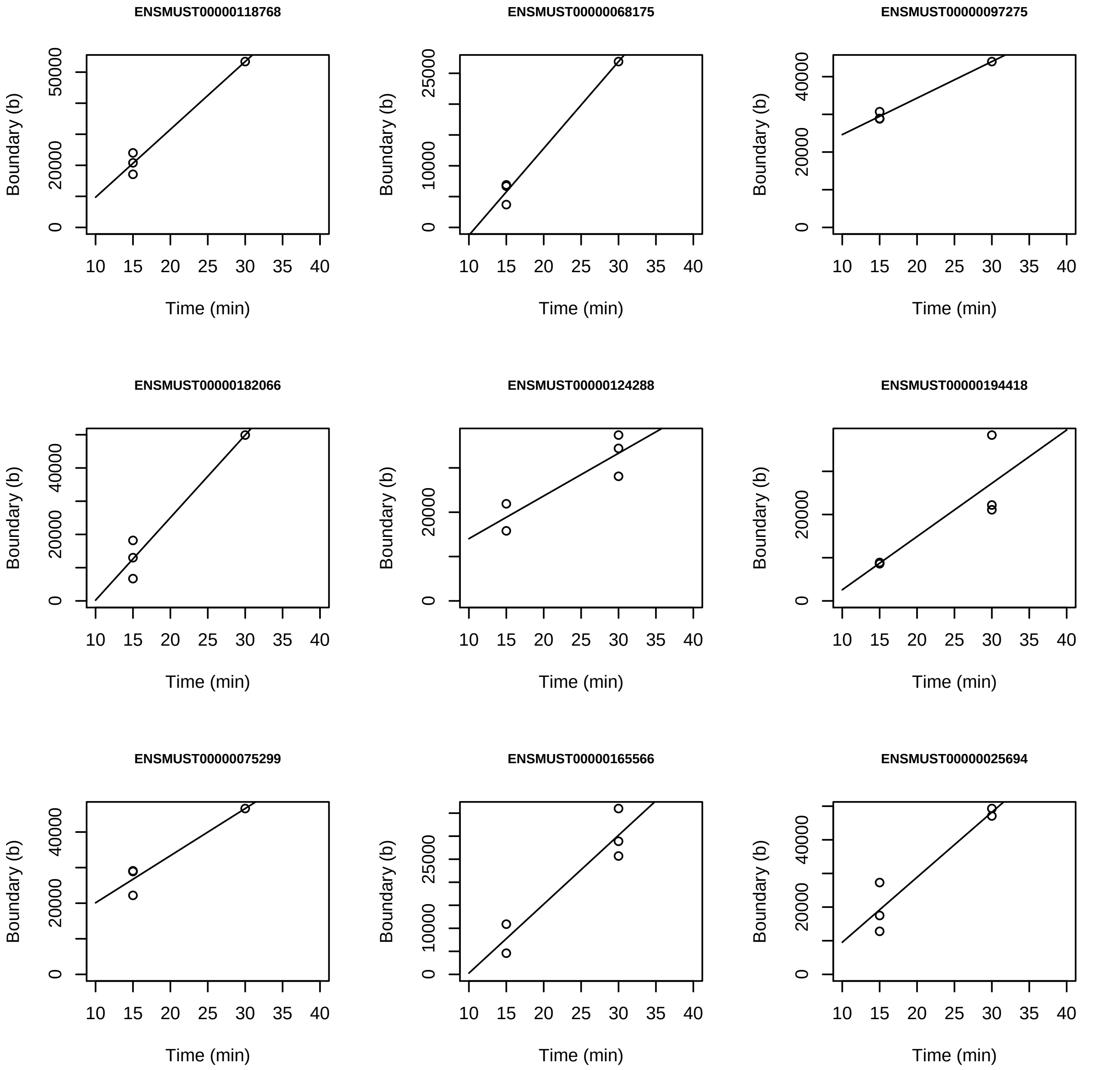
ENSMUST00000118768
ENSMUST00000068175
ENSMUST00000097275
ENSMUST00000182066
ENSMUST00000124288
ENSMUST00000194418
ENSMUST00000075299
ENSMUST00000165566
ENSMUST00000025694
Boundary (b)
Time (min)
10 15 20 25 30 35 40
0
10000
20000
25000
40000
50000

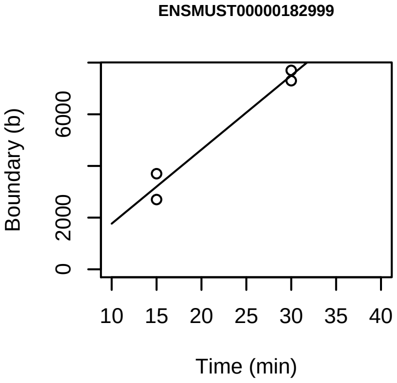
ENSMUST00000182999
Boundary (b)
0
2000
6000
10
15
20
25
30
35
40
Time (min)

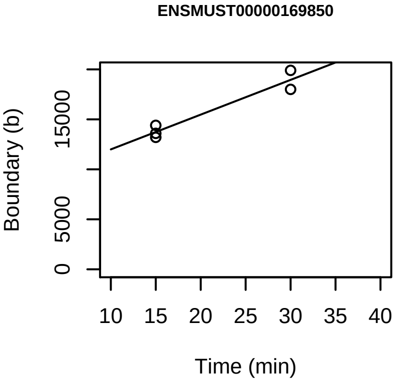
ENSMUST00000169850
Boundary (b)
0
5000
15000
10
15
20
25
30
35
40
Time (min)
