# Supplementary material for: A slow transcription rate causes embryonic lethality and perturbs kinetic coupling of neuronal genes
Source: EMBO J. 2019 Apr 15;38(9):e101244. doi: 10.15252/embj.2018101244 (PMC6484407; doi:10.15252/embj.2018101244)
Supplement: Supplementary file 8 — Source Data for Figure 3 [file EMBJ-38-e101244-s007.zip › Source_Data_Fig3_wt.pdf]

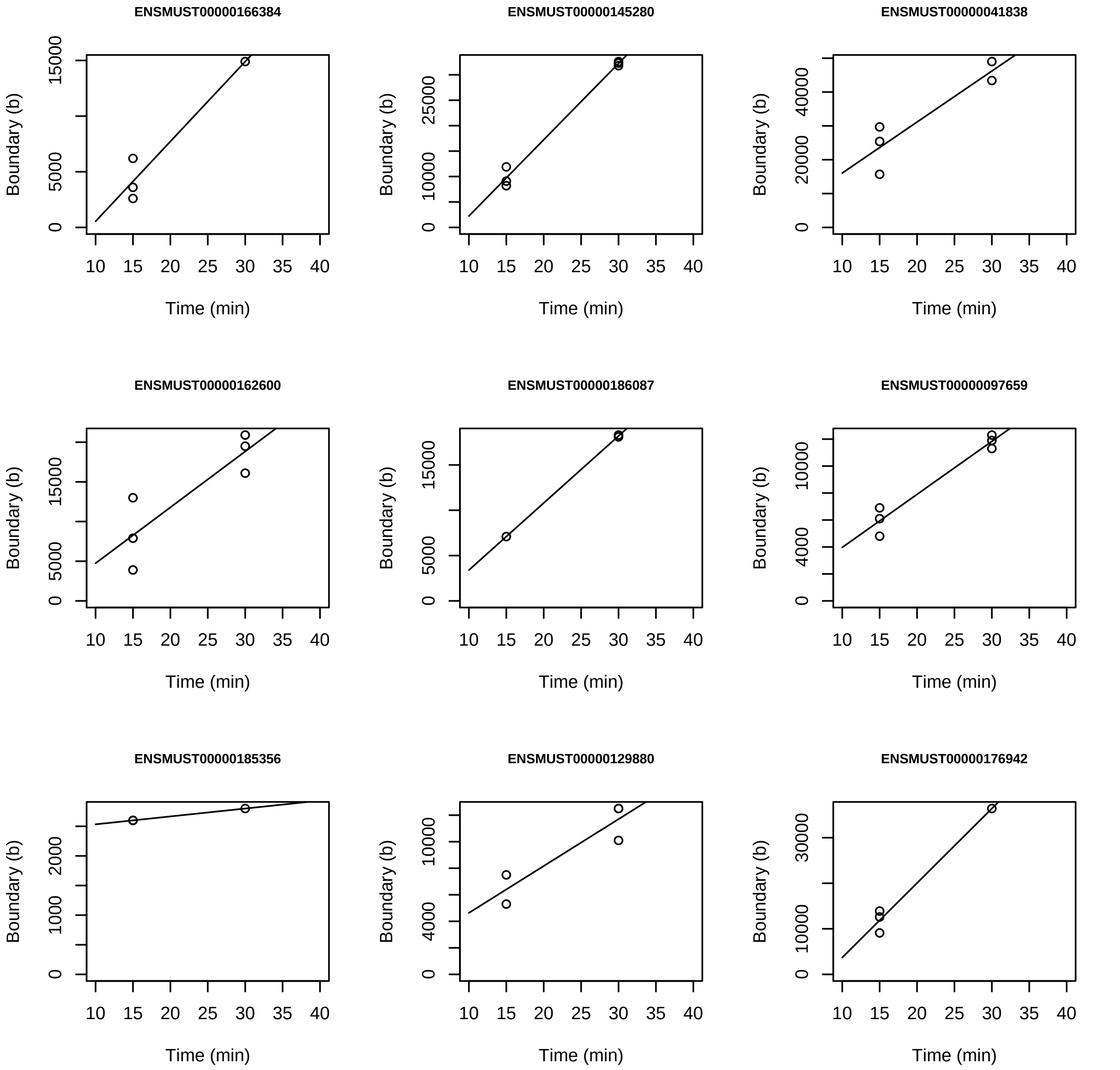

ENSMUST00000166384
ENSMUST00000145280
ENSMUST00000041838
ENSMUST00000162600
ENSMUST00000186087
ENSMUST00000097659
ENSMUST00000185356
ENSMUST00000129880
ENSMUST00000176942
Boundary (b)
Time (min)
10 15 20 25 30 35 40
0 5000 15000
0 10000 25000
0 20000 40000
0 5000 15000
0 5000 15000
0 4000 10000
0 1000 2000
0 4000 10000
0 10000 30000

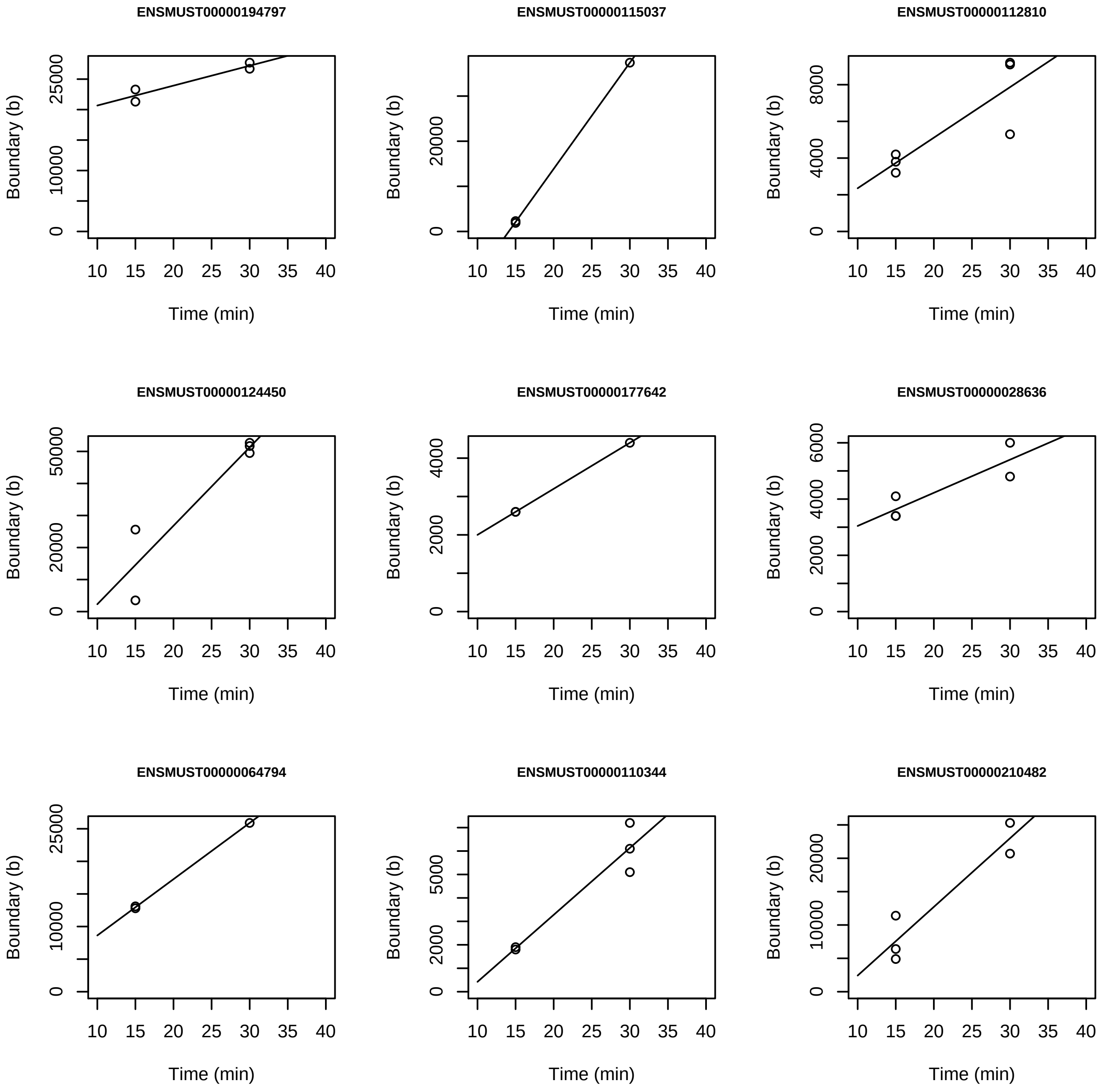
ENSMUST00000194797
ENSMUST00000115037
ENSMUST00000112810
ENSMUST00000124450
ENSMUST00000177642
ENSMUST00000028636
ENSMUST00000064794
ENSMUST00000110344
ENSMUST00000210482
Boundary (b)
Time (min)
10 15 20 25 30 35 40
0
10000
25000
20000
4000
8000
50000
2000
6000
5000

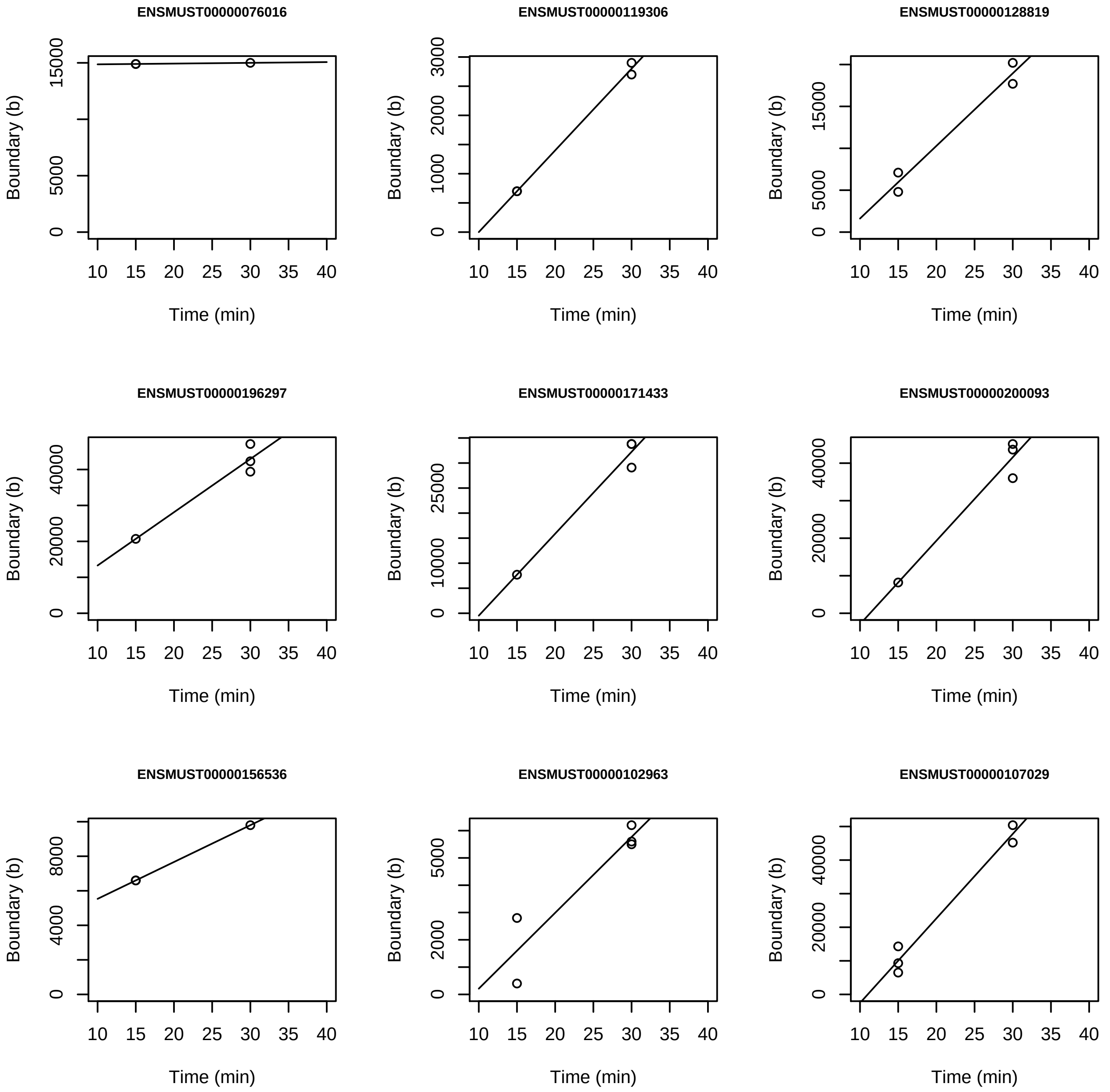
ENSMUST00000076016
Boundary (b)
0
5000
15000
Time (min)
10 15 20 25 30 35 40
ENSMUST00000119306
Boundary (b)
0
1000
2000
3000
Time (min)
10 15 20 25 30 35 40
ENSMUST00000128819
Boundary (b)
0
5000
15000
Time (min)
10 15 20 25 30 35 40
ENSMUST00000196297
Boundary (b)
0
20000
40000
Time (min)
10 15 20 25 30 35 40
ENSMUST00000171433
Boundary (b)
0
10000
25000
Time (min)
10 15 20 25 30 35 40
ENSMUST00000200093
Boundary (b)
0
20000
40000
Time (min)
10 15 20 25 30 35 40
ENSMUST00000156536
Boundary (b)
0
4000
8000
Time (min)
10 15 20 25 30 35 40
ENSMUST00000102963
Boundary (b)
0
2000
5000
Time (min)
10 15 20 25 30 35 40
ENSMUST00000107029
Boundary (b)
0
20000
40000
Time (min)
10 15 20 25 30 35 40

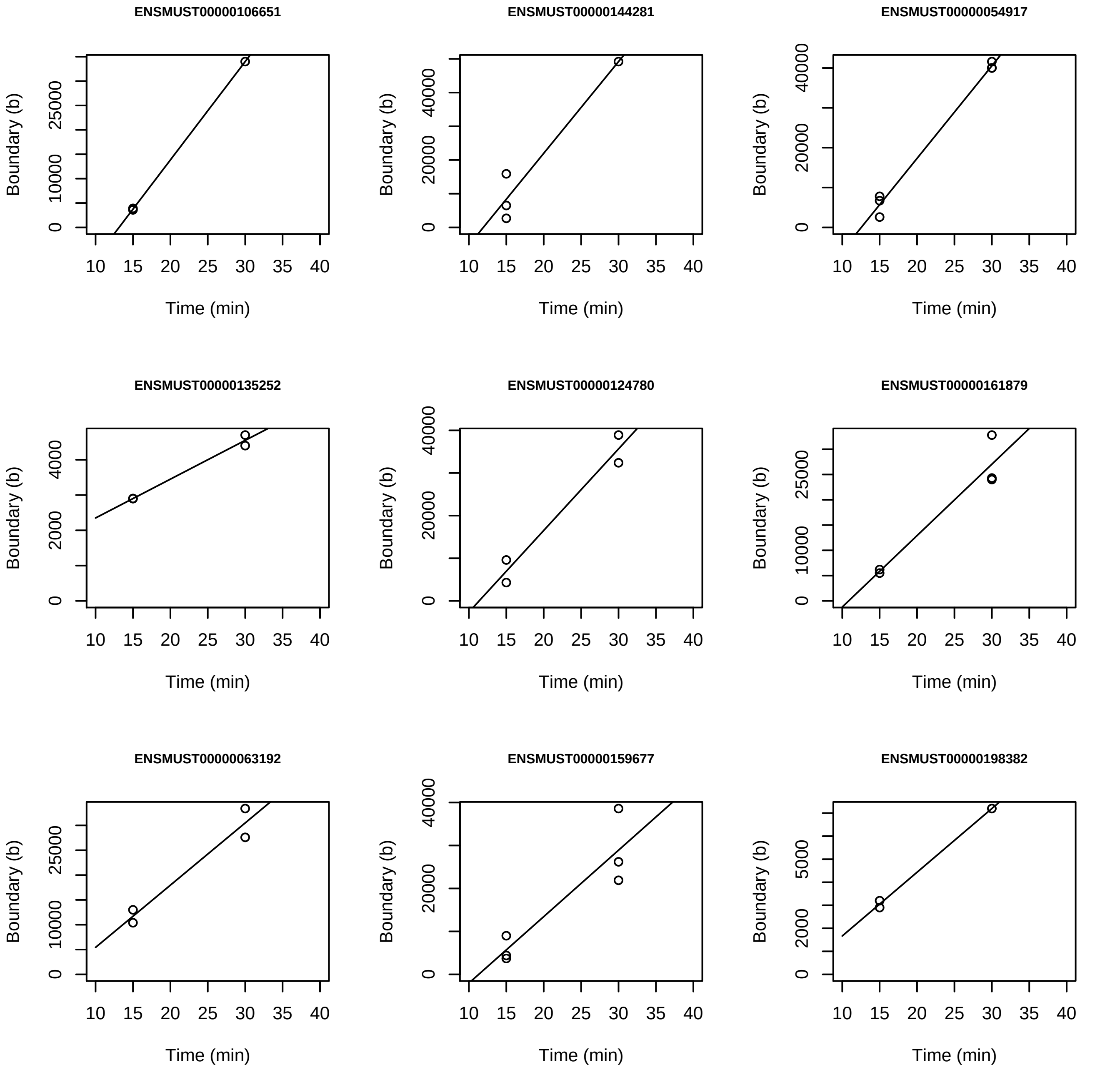
ENSMUST00000106651
ENSMUST00000144281
ENSMUST00000054917
ENSMUST00000135252
ENSMUST00000124780
ENSMUST00000161879
ENSMUST00000063192
ENSMUST00000159677
ENSMUST00000198382
Boundary (b)
Time (min)

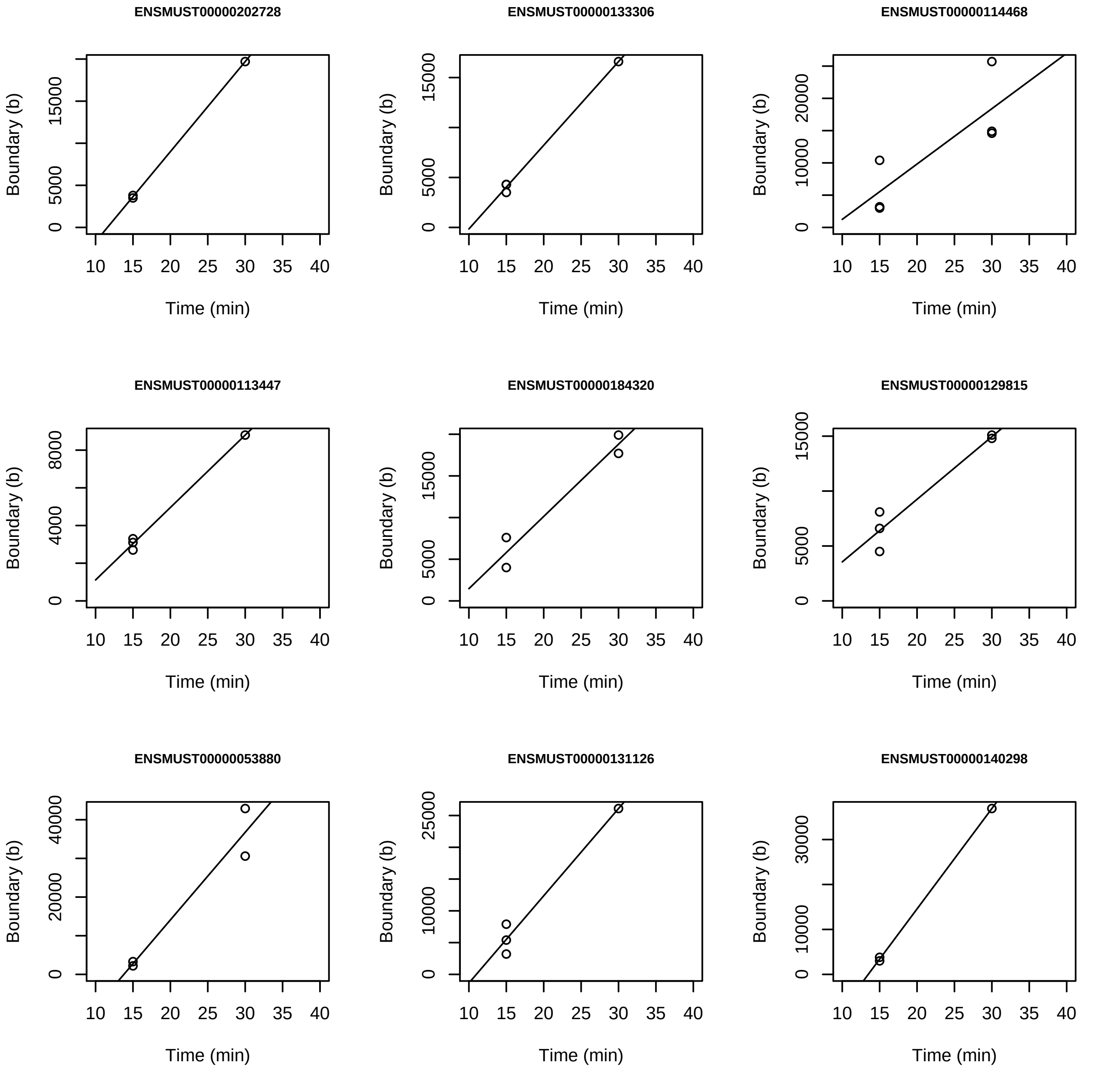
ENSMUST00000202728
Boundary (b)
0
5000
15000
Time (min)
10
15
20
25
30
35
40
ENSMUST00000133306
Boundary (b)
0
5000
15000
Time (min)
ENSMUST00000114468
Boundary (b)
0
10000
20000
Time (min)
ENSMUST00000113447
Boundary (b)
0
4000
8000
Time (min)
ENSMUST00000184320
Boundary (b)
0
5000
15000
Time (min)
ENSMUST00000129815
Boundary (b)
0
5000
15000
Time (min)
ENSMUST00000053880
Boundary (b)
0
20000
40000
Time (min)
ENSMUST00000131126
Boundary (b)
0
10000
25000
Time (min)
ENSMUST00000140298
Boundary (b)
0
10000
30000
Time (min)

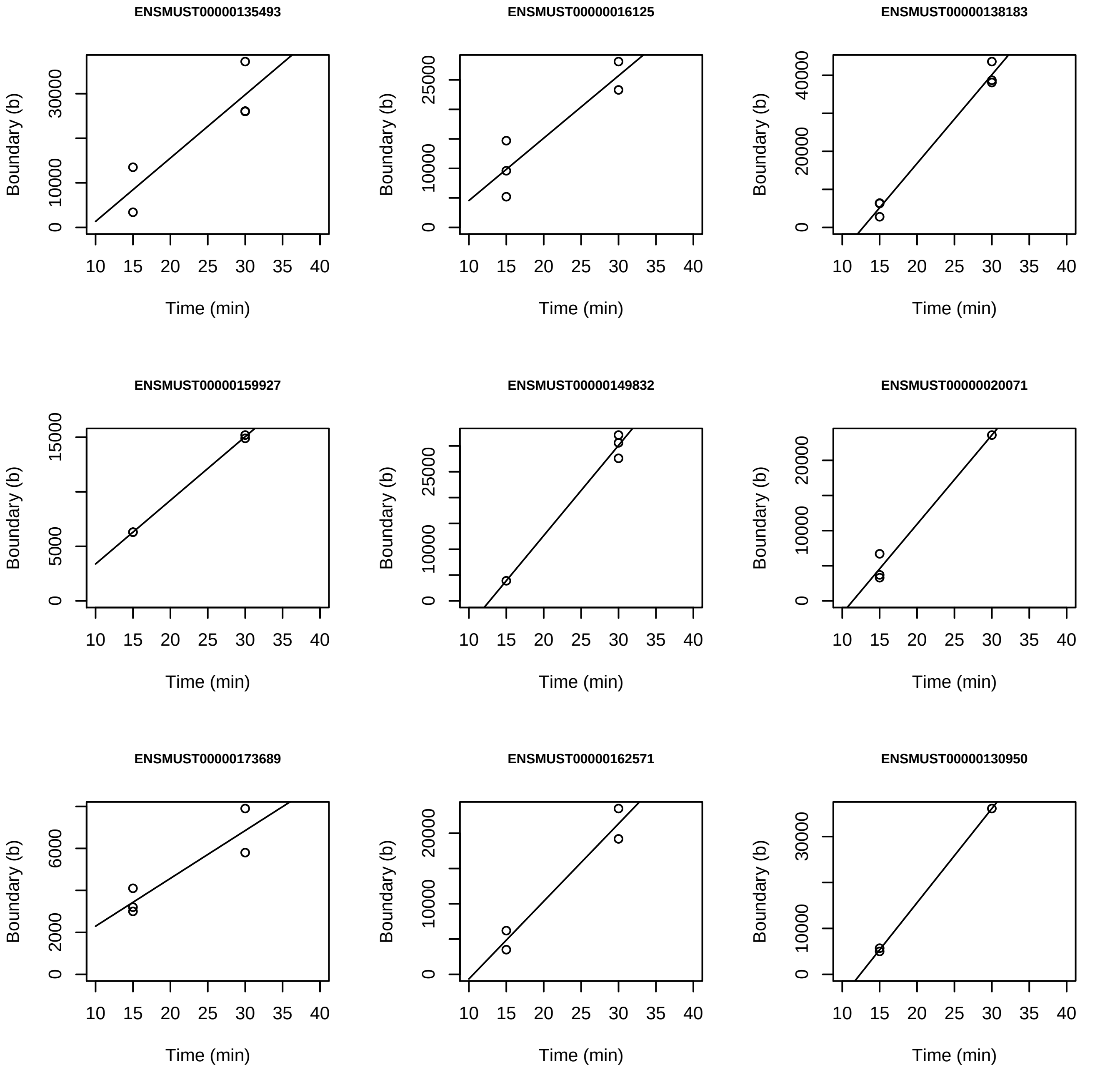
ENSMUST00000135493
ENSMUST00000016125
ENSMUST00000138183
ENSMUST00000159927
ENSMUST00000149832
ENSMUST00000020071
ENSMUST00000173689
ENSMUST00000162571
ENSMUST00000130950
Boundary (b)
Time (min)
10 15 20 25 30 35 40
0 10000 30000
0 10000 25000
0 20000 40000
0 5000 15000
0 10000 25000
0 10000 20000
0 2000 6000
0 10000 20000
0 10000 30000

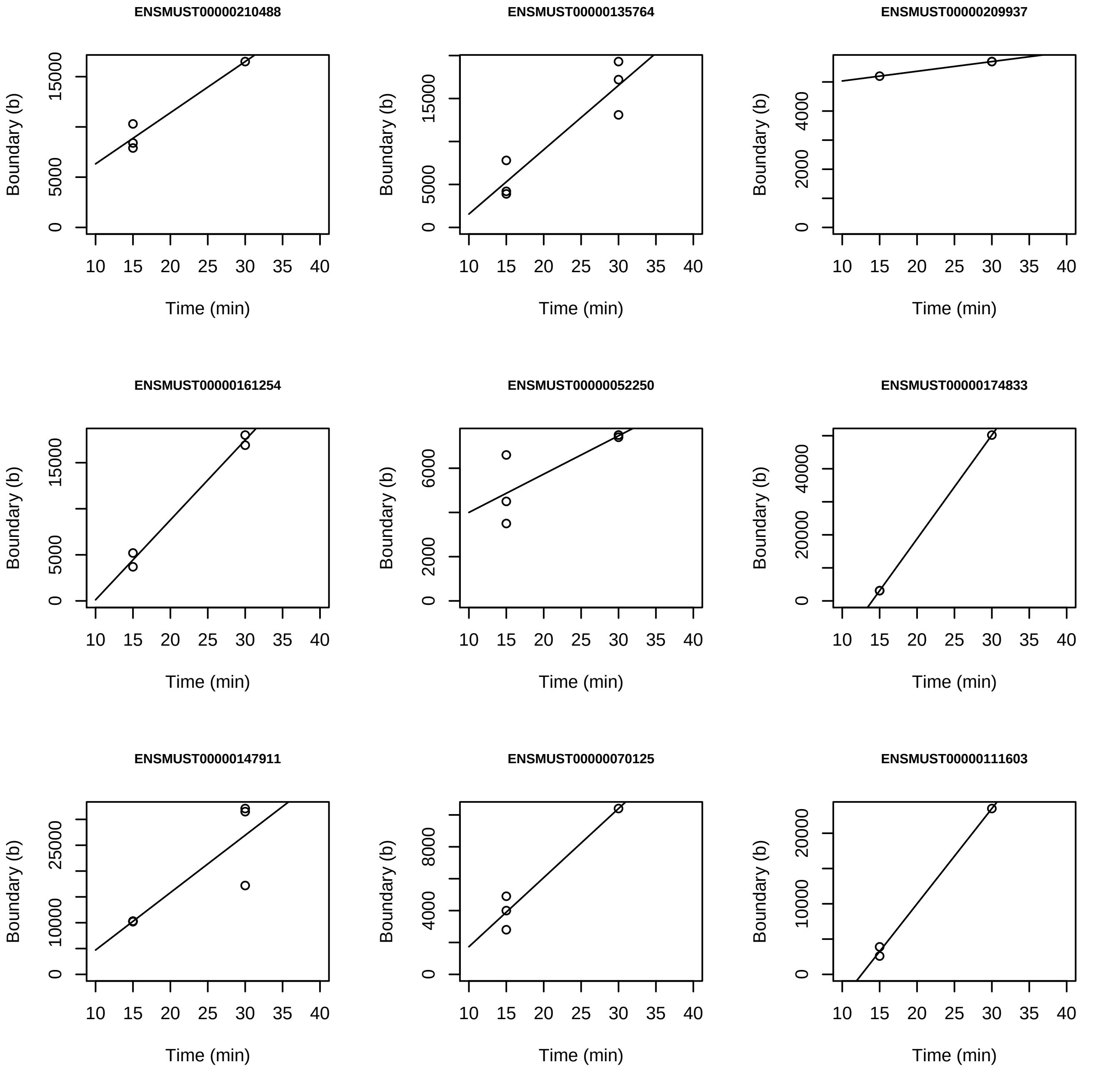
ENSMUST00000210488
Boundary (b)
0
5000
15000
Time (min)
10
15
20
25
30
35
40
ENSMUST00000135764
Boundary (b)
0
5000
15000
Time (min)
ENSMUST00000209937
Boundary (b)
0
2000
4000
Time (min)
ENSMUST00000161254
Boundary (b)
0
5000
15000
Time (min)
ENSMUST00000052250
Boundary (b)
0
2000
6000
Time (min)
ENSMUST00000174833
Boundary (b)
0
20000
40000
Time (min)
ENSMUST00000147911
Boundary (b)
0
10000
25000
Time (min)
ENSMUST00000070125
Boundary (b)
0
4000
8000
Time (min)
ENSMUST00000111603
Boundary (b)
0
10000
20000
Time (min)

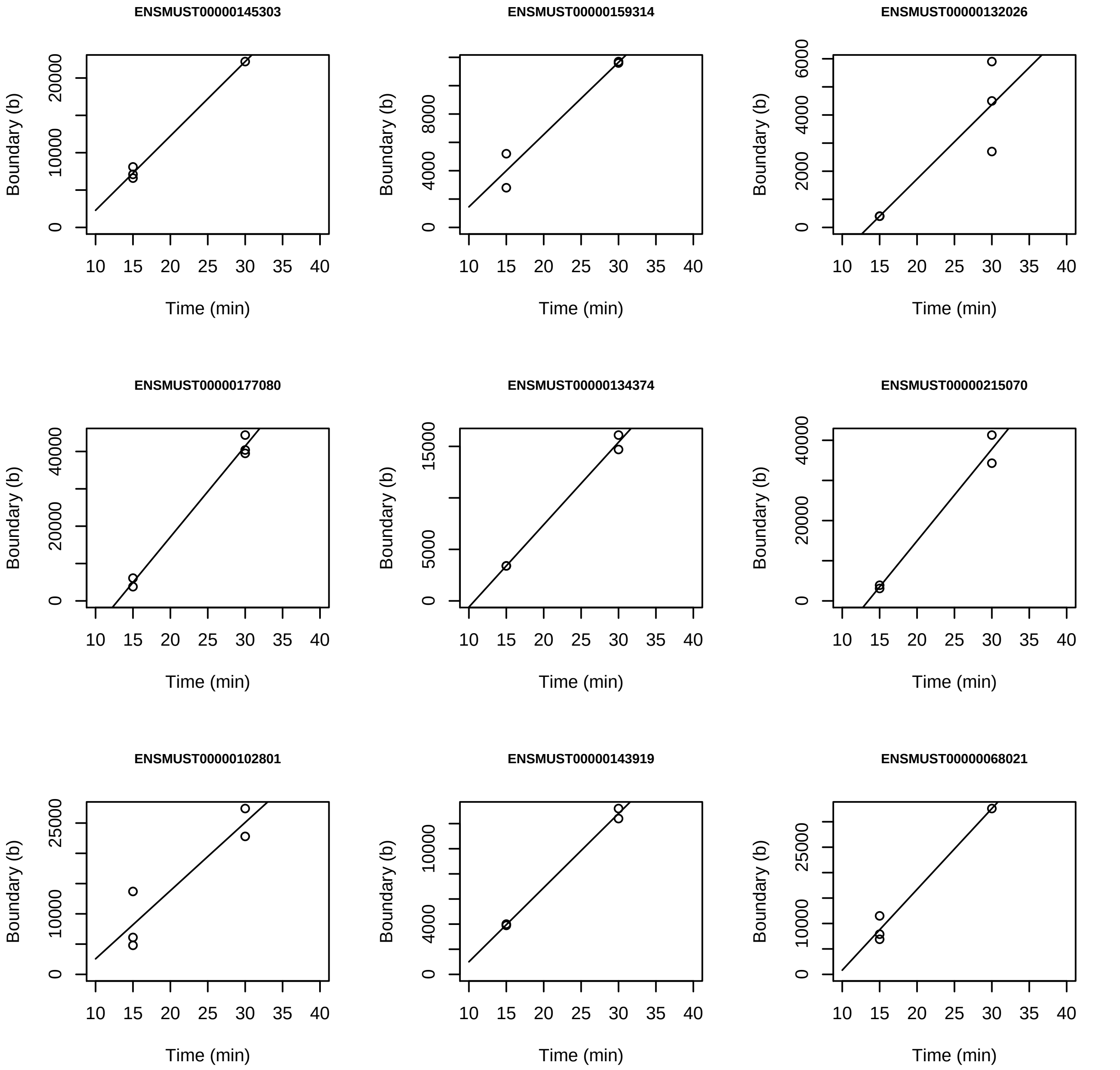

ENSMUST00000145303
Boundary (b)
0
10000
20000
Time (min)
10
15
20
25
30
35
40
ENSMUST00000159314
Boundary (b)
0
4000
8000
Time (min)
10
15
20
25
30
35
40
ENSMUST00000132026
Boundary (b)
0
2000
4000
6000
Time (min)
10
15
20
25
30
35
40
ENSMUST00000177080
Boundary (b)
0
20000
40000
Time (min)
10
15
20
25
30
35
40
ENSMUST00000134374
Boundary (b)
0
5000
15000
Time (min)
10
15
20
25
30
35
40
ENSMUST00000215070
Boundary (b)
0
20000
40000
Time (min)
10
15
20
25
30
35
40
ENSMUST00000102801
Boundary (b)
0
10000
25000
Time (min)
10
15
20
25
30
35
40
ENSMUST00000143919
Boundary (b)
0
4000
10000
Time (min)
10
15
20
25
30
35
40
ENSMUST00000068021
Boundary (b)
0
10000
25000
Time (min)
10
15
20
25
30
35
40

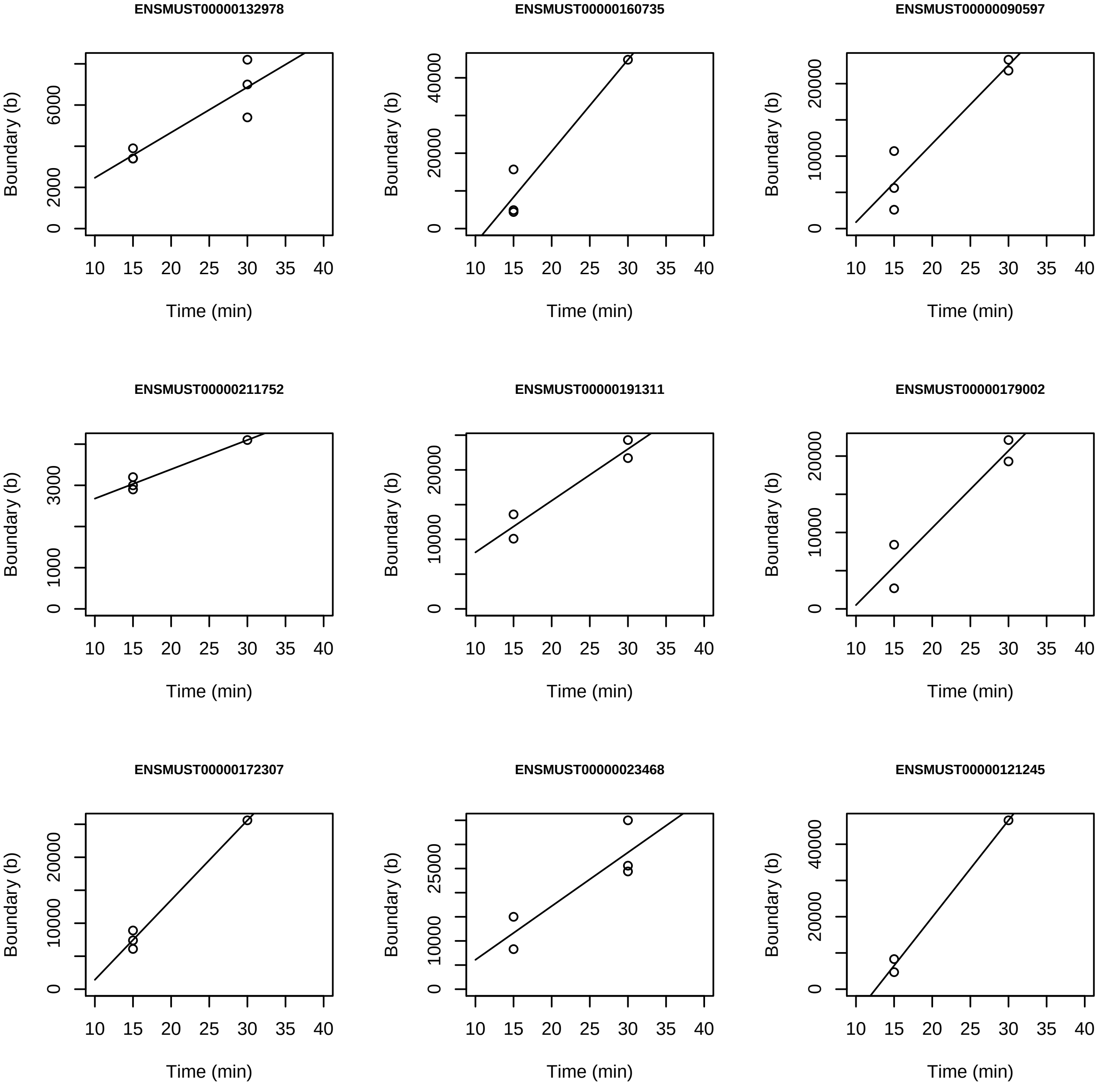
ENSMUST00000132978
Boundary (b)
0
2000
6000
Time (min)
10
15
20
25
30
35
40
ENSMUST00000160735
Boundary (b)
0
20000
40000
Time (min)
10
15
20
25
30
35
40
ENSMUST00000090597
Boundary (b)
0
10000
20000
Time (min)
10
15
20
25
30
35
40
ENSMUST00000211752
Boundary (b)
0
1000
3000
Time (min)
10
15
20
25
30
35
40
ENSMUST00000191311
Boundary (b)
0
10000
20000
Time (min)
10
15
20
25
30
35
40
ENSMUST00000179002
Boundary (b)
0
10000
20000
Time (min)
10
15
20
25
30
35
40
ENSMUST00000172307
Boundary (b)
0
10000
20000
Time (min)
10
15
20
25
30
35
40
ENSMUST00000023468
Boundary (b)
0
10000
25000
Time (min)
10
15
20
25
30
35
40
ENSMUST00000121245
Boundary (b)
0
20000
40000
Time (min)
10
15
20
25
30
35
40

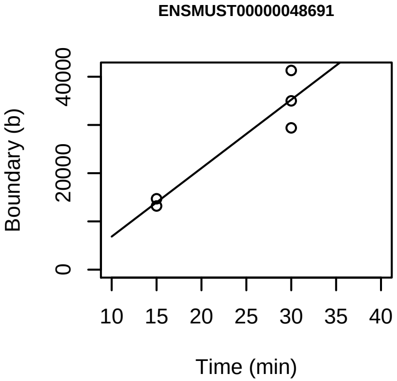
ENSMUST00000048691
Boundary (b)
0
20000
40000
10
15
20
25
30
35
40
Time (min)

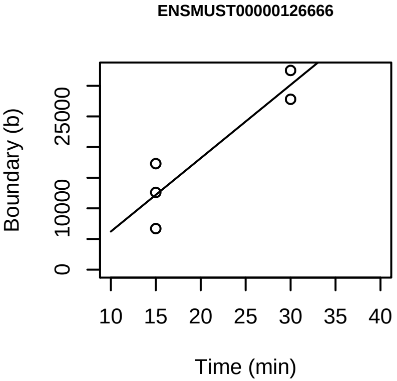
ENSMUST00000126666
Boundary (b)
0
10000
25000
10
15
20
25
30
35
40
Time (min)
