# Supplementary figures and images for: A slow transcription rate causes embryonic lethality and perturbs kinetic coupling of neuronal genes
Source: EMBO J. 2019 Apr 15;38(9):e101244. doi: 10.15252/embj.2018101244 (PMC6484407; doi:10.15252/embj.2018101244)

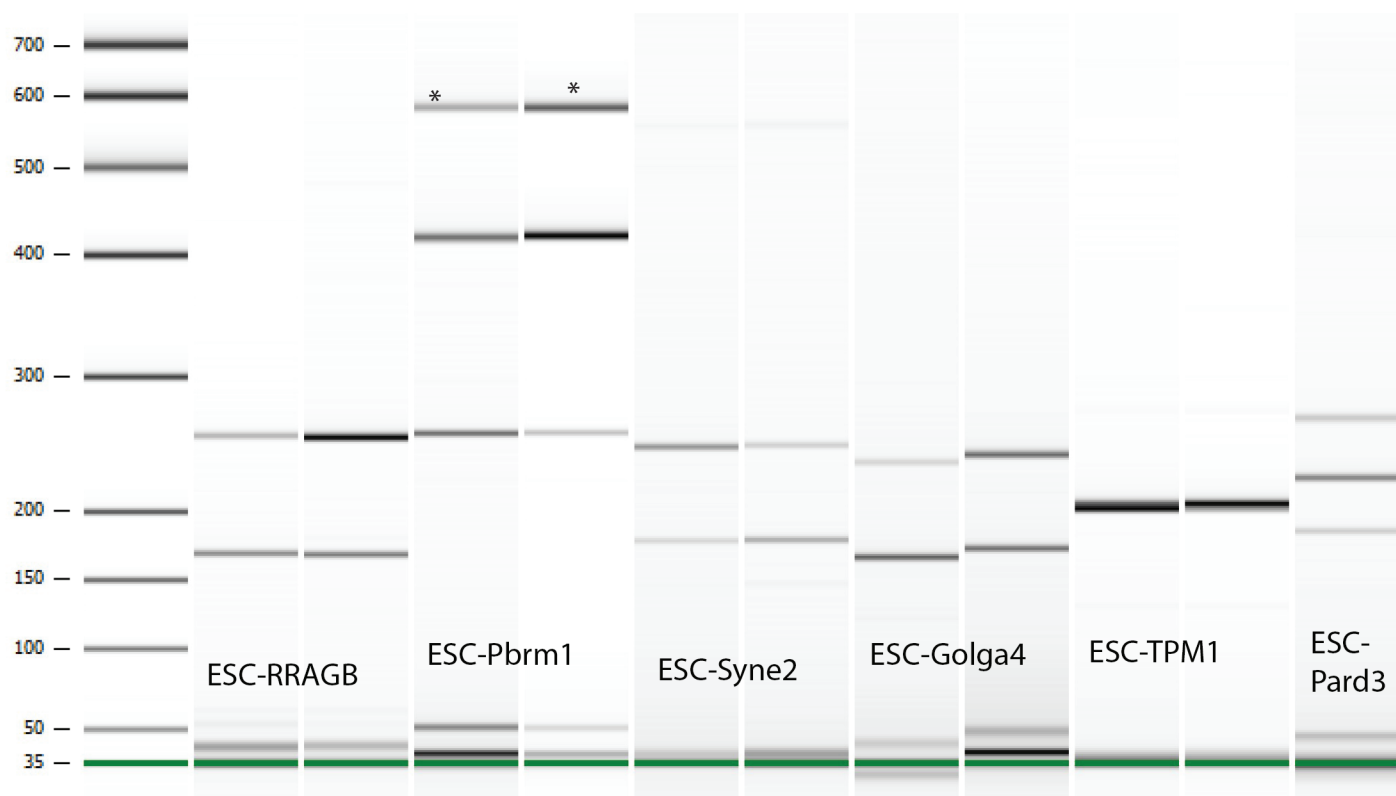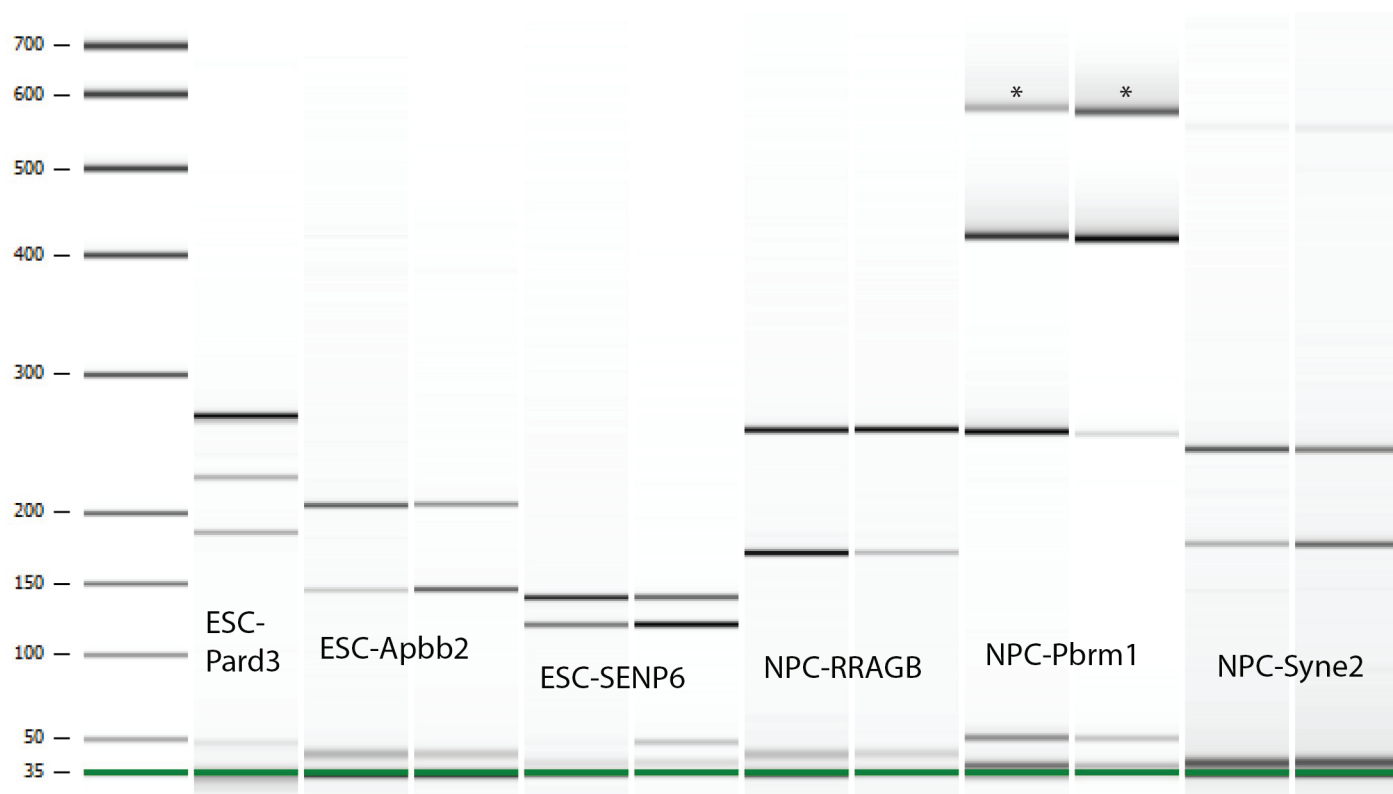

Supplement: Supplementary file 9 — Source Data for Figure 5 [file EMBJ-38-e101244-s008.pdf]
